# Open innovation: status quo and quo vadis - an analysis of a research field

**DOI:** 10.1007/s11846-023-00655-8

**Published:** 2023-03-25

**Authors:** Alberto Bertello, Paola De Bernardi, Francesca Ricciardi

**Affiliations:** Department of Management, University of Turin, Corso Unione Sovietica 218bis, Turin, Italy

**Keywords:** Open innovation, Bibliometric analysis, Content analysis, Sustainability, Digital transformation, Research agenda

## Abstract

Open innovation is now a widely used concept in academia, industry, and policy-making. According to the recent report “The Open Innovation Barometer”, released by the Economist, 90% of organizations have either adopted or are planning to implement key open innovation practices by opening up their organizational boundaries to collaborative innovation in the next three years (The Economist Group 2022). However, the social and economic changes imposed by the emerging processes of transition towards a more digital and sustainable society raise questions on how the open innovation field of studies is evolving to meet new, emerging needs. By combining bibliometric techniques and content analysis, this study illustrates how this research community has evolved in the last 12 years. More specifically, this study provides a descriptive analysis of the literature on open innovation, defines its knowledge structure, and illustrates a representative picture of the theoretical landscape. Our analysis shows that attempts to consolidate established topics and theoretical approaches in this field of studies go hand in hand with the emergence of new conversations about unexplored dimensions of open innovation. We conclude this article by outlining some avenues for future research on how to conceptualize, theorize, and research (methods and analytical techniques) open innovation.

## Introduction

Defined by Chesbrough and Bogers as a ‘distributed innovation process based on purposively managed knowledge flows across organizational boundaries’ (Chesbrough and Bogers 2014, p. 17), open innovation has revealed a different way of organizing and producing innovation. According to this model, firms cannot innovate efficiently and effectively only from within (Chesbrough 2003). More specifically, superior innovation performance does not exclusively rely on the activity of in-house, vertically integrated R&D laboratories, but rather requires coordination across more disintegrated networks of innovation that connect firms with suppliers, customers, universities, research labs, consultants, and start-up firms (Chesbrough and Bogers 2014; West and Bogers 2017). Almost twenty years of research on open innovation have provided thorough insights into how distributed innovation processes influence firm performance and contribute to speeding up innovation and developing new products (e.g., Dodgson et al. 2006), services (e.g., Chesbrough 2010), and processes (e.g., Bertello et al. 2022a).

Ongoing transition processes towards a more sustainable and digital society are influencing and are in turn being influenced by open innovation practices. The complexity and systemic nature of today’s societal challenges can, in fact, only be addressed by unleashing collective, multi-stakeholder action (George et al. 2016). Moreover, emerging technologies, such as digital platforms and big data analytics, can contribute to build more resilient innovation ecosystems (Bogers et al. 2018a), although they challenge organizations by requiring them to develop specific resources, skills and competencies. This ever-changing scenario paves the way for many reflections. Notwithstanding, as the overarching question of our study, we aimed to explore how the open innovation community of scholars has developed its discourse over the last few years. Therefore, we employed bibliometric techniques, followed by a content analysis of the most relevant articles in the field. By doing so, we were able to address four main research questions:

RQ1. What are the most notable and influential contributions/contributors to open innovation literature?

RQ2. What are the different clusters that emerge from open innovation literature?

RQ3. What is the theoretical landscape that emerges from open innovation literature?

RQ4. What are the research opportunities that might advance research on open innovation?

To answer these research questions, we have articulated our analysis across four steps. First, we developed a descriptive analysis of the sample papers. Second, we conducted a keyword co-occurrence analysis to investigate the knowledge structure of open innovation research. Third, we conducted a network analysis to identify the most relevant theories and the theoretical approaches used in open innovation studies. Fourth, drawing inspiration from the bibliometric and content analysis, we have developed recommendations to advance research in the field of open innovation studies. The overall analysis has been conducted by identifying two time periods (2010‒2015 and 2016‒2021, respectively) to identify how transition processes towards sustainability and digitalization impacted the open innovation research landscape.

Existing reviews of open innovation research have principally developed reviews that either privileged an all-comprehensive yet rather static and atemporal overview of this research domain (e.g., West and Bogers 2014) or focused on a specific domain such as knowledge management (e.g., Natalicchio et al. 2017) or sustainability (e.g., Kimpimäki et al. 2022). The most relevant attempt to explore open innovation research holistically, comparing early and current research, dates back to 2016, when Randhawa and colleagues compared the time periods 2003‒2008 and 2009‒2013 (Randhawa et al. 2016). Our article re-proposes and extends this analytical approach with the twofold aim of outlining fresher insights about the historical evolution of this research community’s outputs and inviting academics and practitioners to (re)think open innovation today.

The integration of our insights with earlier literature reviews in the open innovation domain suggests that open innovation research has developed around three phases so far. A first phase, though not the object of our analysis, saw a few scholars leading the field and introducing successful descriptive case studies of early adopters such as big corporates and high-tech start-ups (Huizingh 2011). In the second phase, new scholars joined the community, riding the wave of ongoing conversations principally framed around the role of users and customers in value co-creation processes, firms’ ability to internalize externally developed knowledge (e.g., inbound open innovation), and the governance principles and strategic implications of open innovation from a firm-centric perspective (e.g., how firms capture value from inter-organizational knowledge flows, and how firms build competitive advantage through open innovation strategies). During this second phase, new methodologies, especially quantitative studies, spread to consolidate the insights developed in earlier research and to test hypotheses drawing on theoretical approaches principally framed in theories of the firm, such as the resource- and knowledge-based views. Finally came a more varied third phase, in which the further consolidation of well-established conversations goes hand in hand with attempts to gradually frame open innovation according to new imperatives. During this phase, scholars have been increasingly highlighting the need to identify not only the bright sides but also the dark sides of open innovation (Chaudhary et al. 2022; Helm et al. 2019; Saura et al. 2023; Stanko et al. 2017), also in light of the growing pressures of local, national, and international policies towards the implementation of open innovation (Bertello et al. 2022a; Marullo et al. 2020). These studies also bring to light reflections about the opportunities and challenges related to the ongoing processes of digital transformation. While some studies have, for instance, highlighted how advanced information systems and emerging technologies such as the Internet of Things, big data analytics, and artificial intelligence facilitate collaboration, exchange of knowledge, and higher innovation performances (e.g., Åström et al. 2022; Del Vecchio et al., 2018; Santoro et al. 2018; Scuotto et al. 2017), others have shifted the attention to the challenges of implementing open innovation in contexts in which the actors involved in the ecosystem, such as public organizations (Scuotto et al. 2016) and SMEs (Bertello et al. 2021a), have limited digital skills and knowledge. An emerging stream of research associating open innovation with a relational ontology (see Emirbayer 1997) has also stimulated reflections of when and how open innovation is convenient and when it is not, shedding light on the unintended consequences of openness (Mair and Gegenhuber 2021) and the positive effects of closure (Diriker et al. 2022; Lauritzen and Karafyllia 2019). Our review also shows how literature streams that have developed separately from open innovation, such as the triple helix (Etzkowitz and Leydesdorff 2000) and social innovation (Murray et al. 2010), have now been converging due to the increasing emphasis that open innovation studies are putting on regional and societal impacts (Bertello et al. 2021a; Santos et al. 2021). Finally, the human side of open innovation seems to have gained much attention over the last years, with increasingly scholarly efforts to explore how human resources, cognition, behavior, and other individual-level attributes influence open innovation practices and performance (e.g., Ahn et al. 2017; Bogers et al. 2018b; Bertello et al. 2022b).

Against this scenario, what is the theoretical landscape that has characterized open innovation research over the last 12 years? Our findings show how in the first time period under analysis (2010‒2015) scholars have principally leveraged on theories of the firm, and, more specifically, the resource-based view and knowledge-based view, to explore how firms obtain and absorb knowledge from the external environment to improve strategically their competitive advantage (e.g., Berchicchi 2013; Brunswicker and Vanhaverbeke 2015; Lee et al. 2010). In the second time period (2016‒2021), the resource-based view and knowledge-based view have further consolidated their predominance, although in the meantime a new constellation of theoretical approaches has emerged. It seems, therefore, that some scholars have tried, on the one hand, to ride the wave of these well-established theories, while others have started renewing the theoretical ground to develop new conversations that respond to the need to capture the complexity of the empirical scenario, complexity that can no longer be explained exclusively by traditional theories of the firm. In light of these reflections, we end our article by outlining avenues for future research that support advances in the field of open innovation studies as concerns concepts, definitions, theories, and methods.

## Methodology

Bibliometrics can be defined as the research field in which the quantitative aspects of bibliographic material are studied (Broadus 1987). It consists of a library and information science research field where bibliographic material is studied using quantitative methods (Broadus 1987; Pritchard 1969). For this reason, scholars have often used the terms informetrics and scientometrics as related approaches (Mas-Tur et al. 2020). In comparison to other research approaches, a bibliometric analysis enables researchers to investigate a larger amount of data than systematic literature reviews, in a more automated and relatively unbiased fashion (Kimpimäki et al. 2022) that ensures high levels of rigor, scientific soundness, transparency, and replicability (Dada et al. 2018; Rey-Martí et al. 2016).

In this study, bibliometric techniques are combined with content analysis (see Kraus et al., 2022). More specifically, the methodology used in this paper consists of three approaches. First, we applied bibliometric methods to perform a descriptive analysis of sample papers by making use of the R-package Bibliometrix tool (Aria and Cuccurullo 2017), a software tool that has been gaining increasing attention among management scholars aiming to perform descriptive analysis starting with bibliographic databases (e.g., Forliano et al. 2021; Secinaro & Calandra 2020). Second, we used the VOSviewer software to visualize the bibliometric clusters emerging from a keyword co-occurrence analysis. Before visualizing the map, however, we normalized the dataset of raw keywords, to reconcile keywords that were referring to the same term but were written in different ways. These were traced back to singular/plural forms, British/American English variants, acronyms, hyphens, and similarities. For example, the keywords ‘R&D’ and ‘research and development’ or ‘business model’ and ‘business models’ occur as different terms but refer to the same meaning; therefore, we reconciled them into a single keyword. We conducted this analysis using OpenRefine (ver. 3.3), an open-source tool originally developed by Google for data-cleaning and successfully employed in several similar studies (see De Bernardi et al. 2021). Considering the size of the database, the different specific algorithms embedded in the software and designated for data reconciliation (https://openrefine.org/) enabled us to obtain more rigorous and replicable results compared to manual analysis. We used this software as it is based on a visualize-and-manipulate paradigm that lets any researcher find networks and data properties easily and effectively. Finally, as a third step of our analysis, we performed a content analysis to support the creation of a network analysis based on the theories and the theoretical approaches used (Ricciardi et al. 2021) in the 30 most relevant articles in the field. This analysis did not rely on the use of any software. Indeed, it was based on reading the 30 manuscripts thoroughly to identify which theoretical lenses were advocated in the papers.

### Data collection and extraction

The first step in a bibliometric analysis is to collect raw data from which the necessary metadata (e.g., authors, number of citations, countries) can be obtained (Carvalho et al. 2013). Several bibliometric databases exist. However, the two largest worldwide-used databases are the Web of Science (WoS) from Clarivate Analytics and Scopus from Elsevier. The WoS contains over 90 million documents from more than 15,000 journals, while Scopus indexes around 69 million records from more than 20,000 active titles (i.e., peer-reviewed journals, books, and conference proceedings) (Forliano et al. 2021). In this study, similarly to bibliometric approaches performed in business and management studies by other scholars (e.g., Forliano et al. 2021; Secinaro & Calandra 2020; Vallaster et al. 2019; Wei et al. 2021), we consulted the WoS Core Collection for conducting the bibliometric analysis. WoS is, in fact, considered a reliable database for data mining and has become one of the primary databases used by scholars for conducting bibliometric analyses (Thelwall 2008; Waltman & van Eck 2012). In line with previous influential bibliometric studies on the same topic (e.g., Randhawa et al. 2016; West & Bogers 2014), we used the search phrase “open innovation” in title, abstract, or keywords. The search was conducted in January 2022. The initial search returned 7534 records. Then, restrictions on the year (from 2010 to 2021 only), document type (articles only, excluding for instance book chapters and proceedings), language (English only), and subject (Business and Management only) were applied, refining the sample to 1998 articles. We also further limited our review to studies in journals ranked 2, 3, 4, or 4* in the Academic Journal Guide/ABS 2021 journals list, as including only top-tier academic journals remains the most frequently adopted method for identifying scholarly debates and research trends when performing the review of specific topic-related literature (Atewologun et al. 2017). The Academic Journal Guide/ABS is not the only journal ranking, but is considered among the major ones (Kraus et al. 2020). Several articles in the field of business and management studies have used this ranking to further refine the search (e.g., Bertello et al. 2022c; Vrontis & Christofi, 2021). Others have used different criteria such as the JCR Impact Factors (IF) by Clarivate Analytics (e.g., Kunisch et al. 2015; Überbacher 2014). Moreover, these different rankings can be compared and converted (Bouncken et al. 2015; Kraus et al. 2020). Based on Kraus et al.’s (2020) conversion table of leading academic journal rankings, a journal ranked ≥ 2 in the Academic Journal Guide/ABS is comparable to a journal ranked ≥ C in the VHB Jourqual (JQ) and ≥ 1.5 in the JCR Impact Factors (IF) by Clarivate Analytics. Based on this information, this criterion allowed us to include in our research the most relevant state-of-the-art knowledge of the topic of interest while keeping the sample manageable. Based on these exclusion/inclusion criteria, our final result was 1515 records. Based on our purpose to explore whether and to what extent the open innovation area of studies is evolving in response to the emerging transition towards a more digital and sustainable society, we set 2015 as a threshold to compare two different time periods (2010‒2015 and 2016‒2021). We followed two criteria to identify the year to be selected as a threshold. We included in our criteria considerations about the impact of digitalization and sustainability issues on scientific production. First, we conducted a preliminary search on the WoS database to explore the research trend of digital transformation studies. We indeed selected the term “digital transformation” and we looked at the distribution of papers per year. The year that shows the greatest increment percentage is 2016, where the number of papers increased from 32 to 146 (+ 456%). 2015, however, represents a turning point also as concerns sustainability issues. In 2015, the United Nations released the Sustainable Development Goals (SDGs), providing a list of 17 goals such as “no poverty” (SDG 1), “good health and well-being” (SDG 3), “decent work and economic growth” (SDG 8), articulated in 169 targets to be achieved by 2030. The SDGs were immediately acknowledged as the translation of grand challenges, raising the awareness of practitioners and scholars that the progress required to achieve these goals involves collective, collaborative, and coordinated efforts (George et al. 2016). They thus represent a relevant turning point to explore potential shifts in business and management scholarly literature (Ricciardi et al. 2021).

**Fig. 1:**
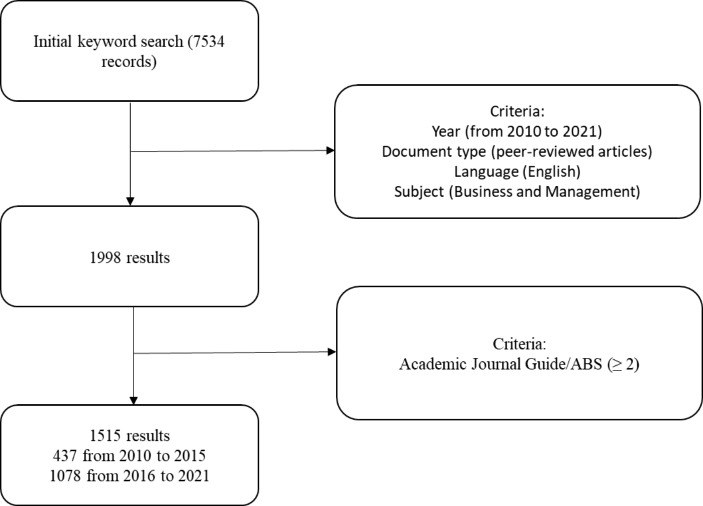
The different phases of the data extraction activity. Source: Authors’ own elaboration

Our paper sample is characterized by 437 papers within the time period 2010‒2015 and 1078 papers within the time period 2016‒2021 (see Fig. 1 for a visual representation of the data extraction activity). Part of our descriptive analysis, as well as the keyword co-occurrence analysis and the content analysis, were structured to compare these two different time periods.

## Descriptive analysis of open innovation studies

In this section, a bibliometric analysis is presented on the basis of different performance indicators. In this way, it is possible to answer the first research question of this study:

What are the most notable and influential contributions/contributors to open innovation literature?

**Fig. 2:**
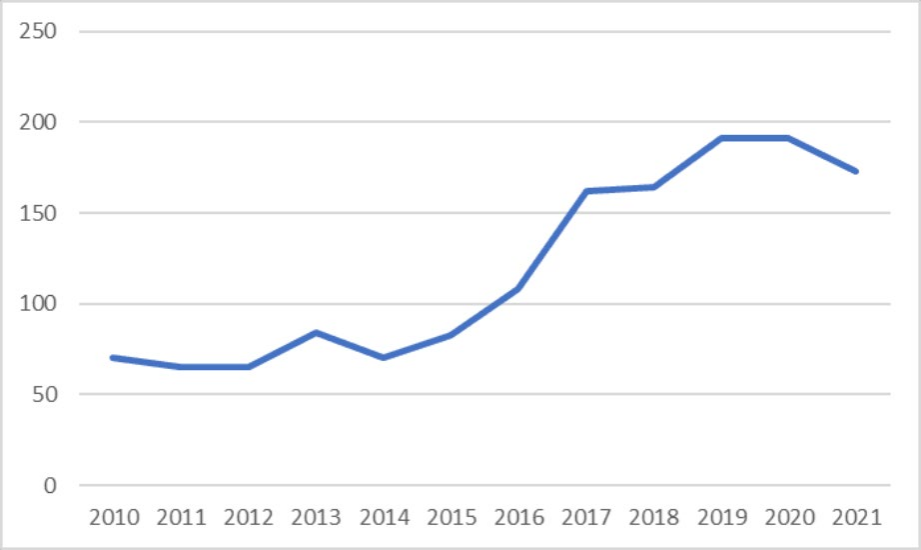
Distribution of publications over time. Source: Authors’ own elaboration.

We therefore conducted a descriptive analysis of our paper sample to outline the articles’ evolution over time, the most influential articles per number of citations, the most productive journals, and the most productive and influential authors. The articles’ evolution within the time period we have selected (i.e., 2010‒2021) shows an incremental growth over the years, with a small decrease in the number of papers published only in the years 2011, 2014, and 2021 (see Fig. 2). The greatest increment was registered in 2017, with an increase from 108 to 162 articles. This means that the production rate from 2016 to 2017 increased by 150%. This extraordinary growth could partially explain why since 2017 the production rate has stabilized, showing only small oscillations (+ 2% in 2018, + 27% in 2019, and − 18% in 2021).

To identify the most influential articles in the open innovation research landscape, we generated two tables to classify the most cited empirical articles in the time periods 2010‒2015 (Table 1) and 2016‒2021 (Table 2), respectively. Citations can synthesize the relevance of a publication in a single number (Forliano et al. 2021), providing a suitable measure to identify the most influential articles (Merigó et al. 2015). More precisely, we included in the tables the total citations received as retrievable in the WoS (TC) and the average number of citations received each year (TCpY). We ranked the articles based on the TCpY index rather than the TC index, since the latter would penalize more recent articles. The most cited empirical paper in the time period 2010‒2015 (i.e., Lee et al. 2010) registers an average of 49.38 citations per year, resulting in a total of 642 citations. Framed in the Korean context, this article introduces stories of OI success indicating networking as one effective way to facilitate OI among SMEs. This paper represents one of the first attempts (an earlier attempt in Van de Vrande et al. 2009 was not included in the time period we considered for our analysis) to discuss open innovation in SMEs, literally opening up a new research stream about open innovation processes, opportunities, and challenges in SMEs (e.g., Bertello et al. 2021a; 2022b; Radziwon et al. 2017; Radziwon & Bogers 2019; Vanhaverbeke 2017). The second most cited article (TCpY: 47.89; TC: 431), co-authored by Laursen and Salter (2014), discusses the paradox of openness. This term indicates the tension between knowledge sharing and knowledge protection that reflects firms’ twofold need to create value through openness while appropriating it through protection. Laursen and Salter’s findings suggest that an appropriability strategy has a concave relationship with external search breadth and collaboration breadth. The third most cited article was published in 2010 by Jeppesen and Lakhani (41.46 TCpY and 539 TC). Although this paper aims to explain individual success in problem-solving, it has been included in the list because Jeppesen and Lakhani’s results contribute to the emerging literature on open and distributed innovation by demonstrating the value of openness in removing barriers to entry to “non-obvious” individuals. In particular, their findings resonate well with recent calls for exploring open innovation models that allow organizations to recombine knowledge with atypical resources (e.g., retired experts, graduate students, and the general public) rather than focusing on the ‘usual suspects’ (Bertello et al. 2021b). Table 1 clearly shows how the most influential journal in terms of highly relevant contributions is Research Policy, which includes 7 out of the 15 most cited publications. This is not surprising, since Research Policy is a multidisciplinary journal devoted to investigating the challenges of various natures posed by innovation. The journal is the only one to be ranked 4* in the innovation field by the Academic Journal Guide/ABS, as well as being one of the first journals to have welcomed studies about open innovation.

**Table 1:** Citation analysis of the 15 most relevant empirical documents in the dataset ordered by the total number of citations received per year (TCpY) in the time period 2010‒2015

Author	Year	Title	Journal	TCpY	TC
Lee, Park, Yoon, and Park	2010	Open innovation in SMEs—An intermediated network model	Research Policy	49.38	642
Laursen and Salter	2014	The paradox of openness: Appropriability, external search and collaboration	Research Policy	47.89	431
Jeppesen and Lakhani	2010	Marginality and Problem-Solving Effectiveness in Broadcast Search	Organization Science	41.46	539
Lee, Hancock, and Hu	2014	Towards an effective framework for building smart cities: Lessons from Seoul and San Francisco	Technological Forecasting and Social Change	34.89	314
Brunswicker and Vanhaverbeke	2015	Open innovation in small and medium-sized enterprises (SMEs): External knowledge sourcing strategies and internal organizational facilitators	Journal of Small Business Management	32.75	262
Parida, Westerberg, and Frishammar	2012	Inbound open innovation activities in high-tech SMEs: the impact on innovation performance	Journal of Small Business Management	31.27	344
Berchicchi	2013	Towards an open R&D system: Internal R&D investment, external knowledge acquisition and innovative performance	Research Policy	30.50	305
Franzoni and Sauermann	2014	Crowd science: The organization of scientific research in open collaborative projects	Research Policy	25.44	229
Spithoven, Vanhaverbeke, and Rojiakkers	2013	Open innovation practices in SMEs and large enterprises	Small Business Economics	24.20	242
Ghisetti, Marzucchi, and Montresor	2015	The open eco-innovation mode. An empirical investigation of eleven European countries	Research Policy	23.37	187
Boudreau	2012	Let a thousand flowers bloom? An early look at large numbers of software app developers and patterns of innovation	Organization Science	22.18	244
Mina, Bascavusoglu-Moreau, and Alan Hughes	2014	Open service innovation and the firm’s search for external knowledge	Research Policy	22	198
Cheng and Huizingh	2014	When is open innovation beneficial? The role of strategic orientation	Journal of Product Innovation Management	20.56	185
Du, Leten, and Vanhaverbeke	2014	Managing open innovation projects with science-based and market-based partners	Research Policy	20.44	184
Bianchi, Cavaliere, Chiaroni, Frattini, and Chiesa	2011	Organisational modes for Open Innovation in the bio-pharmaceutical industry: An exploratory analysis	Technovation	19.92	239

Source: Authors’ own elaboration

**Table 2:** Citation analysis of the 15 most relevant empirical documents in the dataset ordered by the total number of citations received per year (TCpY) in the time period 2016‒2021

Author	Year	Title	Journal	TCpY	TC
Chesbrough	2020	To recover faster from Covid-19, open up: Managerial implications from an open innovation perspective.	Industrial Marketing Management	38	114
Santoro, Vrontis, Thrassou, and Dezi	2018	The Internet of Things: Building a knowledge management system for open innovation and knowledge management capacity	Technological Forecasting and Social Change	37.8	189
Papa, Dezi, Gregori, Mueller, & Miglietta	2018	Improving innovation performance through knowledge acquisition: the moderating role of employee retention and human resource management practices.	Journal of Knowledge Management	35.33	106
Singh, Gupta, Busso, & Kamboj	2021	Top management knowledge value, knowledge sharing practices, open innovation and organizational performance	Journal of Business Research	34.50	69
Ferraris, Santoro, and Dezi	2017	How MNC’s subsidiaries may improve their innovative performance? The role of external sources and knowledge management capabilities	Journal of Knowledge Management	30.17	181
Vrontis, Thrassou, Santoro, and Papa	2017	Ambidexterity, external knowledge and performance in knowledge-intensive firms	The Journal of Technology Transfer	28.17	169
Scuotto, Del Giudice, Bresciani, and Meissner	2017	Knowledge-driven preferences in informal inbound open innovation modes. An explorative view on small to medium enterprises	Journal of Knowledge Management	26.33	158
Martinez-Conesa, Soto-Acosta, and Carayannis	2017	On the path towards open innovation: Assessing the role of knowledge management capability and environmental dynamism in SMEs	Journal of Knowledge Management	25.50	153
Scuotto, Santoro, Bresciani, and Del Giudice	2017	Shifting intra-and inter‐organizational innovation processes towards digital business: an empirical analysis of SMEs.	Creativity and Innovation Management	24.67	148
Parker and Van Alstyne	2018	Innovation, openness, and platform Control	Management Science	24.20	121
Popa, Soto-Acosta, and Martinez- Conesa	2017	Antecedents, moderators, and outcomes of innovation climate and open innovation: An empirical study in SMEs	Technological Forecasting and Social Change	22.67	136
Bogers, Foss, and Lyngsie	2018	The “human side” of open innovation: The role of employee diversity in firm-level openness	Research Policy	22	110
Flor, Cooper, and Oltra	2018	External knowledge search, absorptive capacity and radical innovation in high- technology firms	European Management Journal	20.20	101
Cassiman and Valentini	2016	Open innovation: are inbound and outbound knowledge flows really complementary?	Strategic Management Journal	19.57	137
Scuotto, Ferraris, and Bresciani	2016	Internet of Things: applications and challenges in smart cities. A case study of IBM smart city projects	Business Process Management Journal	18	126

Source: Authors’ own elaboration

The analysis of the 15 most cited empirical papers in the time period 2016‒2021 reveals a different scenario. First of all, the distribution of journals is completely different. Research Policy only counts one paper, while other new journals have emerged. The Journal of Knowledge Management recurs four times in this list, as if to prove the increasing attention that open innovation scholars have devoted to organizational learning and knowledge management processes. The greater distribution of papers across different journals (only Journal of Knowledge Management and Technological Forecasting and Social Change count more than 1 publication in the list) suggests that open innovation research is a hot topic that is no longer exclusively managed by a few leading journals in the innovation field. At the top of the list, with an index of 38 TCpY and 114 TC is not surprisingly the paper that Chesbrough has recently published to highlight the role of open innovation in response to COVID-19. Although the paper does not draw on primary data, we included it in the list since it does provide empirical examples of how open innovation was adopted in medical science to fight COVID-19. The second most influential paper in the time period 2016‒2021 has been co-authored by Santoro, Vrontis, Thrassou, and Dezi. The paper, published in 2018 in the Technological Forecasting and Social Change journal, reflects on the implications of the Internet of Things in the field of knowledge management. More specifically, the authors found how the Internet of Things offers firms new opportunities to develop knowledge management systems that facilitate the creation of open and collaborative ecosystems and the exploitation of internal and external knowledge. The third paper per number of citations has been written by Papa, Dezi, Gregori, Mueller, and Miglietta and published in the Journal of Knowledge Management in 2020^1^. This paper mainly contributes to the literature on open innovation and knowledge management by studying the relationship between knowledge acquisition and innovation performance, and the moderating effects of human resource management, in terms of employee retention and human resource management practices, on the above-mentioned relationship.

Tables 1 and 2 have partially shown how open innovation articles have been published in a greater variety of journals in the time period 2016‒2021 compared to 2010‒2015. To explore which journals have contributed to further spread research on open innovation, we have identified the most productive journals for both periods, and ordered them by the number of open innovation articles published (Tables 3 and 4). In this regard, the most productive journals between 2010 and 2015 are the International Journal of Technology Management, Research Policy, and Technovation, with 37, 32, and 30 articles, respectively.

**Table 3:** The 10 most relevant journals ordered by the number of contributions to the open innovation field of studies in the time period 2010‒2015

Journal	N. Articles	ABS field
International Journal of Technology Management	37	Operations and technology management
Research Policy	32	Innovation
Technovation	30	Innovation
Research-Technology Management	29	Innovation
Technology Analysis & Strategic Management	27	Strategy
R&D Management Journal	26	Innovation
Journal of Product Innovation Management	20	Innovation
Creativity and Innovation Management	19	Innovation
Technological Forecasting and Social Change	18	Innovation
California Management Review16General	16	General Management, Ethics, Gender and Social Responsibility

Source: Authors’ own elaboration

**Table 4:** The 10 most relevant journals ordered by the number of contributions to the open innovation field of studies in the time period 2016‒2021

Journal	N. Articles	ABS field
Technological Forecasting and Social Change	89	Innovation
International Journal of Innovation Management	57	Innovation
Journal of Knowledge Management	52	Organizational Studies
R&D Management Journal	52	Innovation
Management Decision	49	General Management, Ethics, Gender and Social Responsibility
Journal of Business Research	44	General Management, Ethics, Gender and Social Responsibility
Technology Analysis and Strategic Management	37	Strategy
Research Policy	36	Innovation
Creativity and Innovation Management	30	Innovation
Business Process Management Journal	25	Operations and technology management

Source: Authors’ own elaboration

The scenario has changed in the subsequent time period, where the number of articles is more conspicuous and new journals have promoted open innovation as a core topic in their research agenda. The most productive journals between 2016 and 2021 have been Technological Forecasting and Social Change (89), the International Journal of Innovation Management (57), and the Journal of Knowledge Management (52). While the International Journal of Innovation Management and the Journal of Knowledge Management were not included in the 2010‒2015 list, the Technological Forecasting and Social Change journal was then only at ninth position with 18 articles. A comparison between the two tables shows that journals whose core topic is innovation (e.g., Research Policy, R&D Management Journal, Creativity and Innovation Management) are still steadily publishing open innovation articles. However, while 7 out of the 10 most productive journals belonged to the field of innovation in the time period 2010‒2015, this number decreased to 5 journals in 2016‒2021. This suggests that open innovation is constantly attracting new journals, increasing also the probability that open innovation is explored from a variety of lenses and disciplines.

**Fig. 3:**
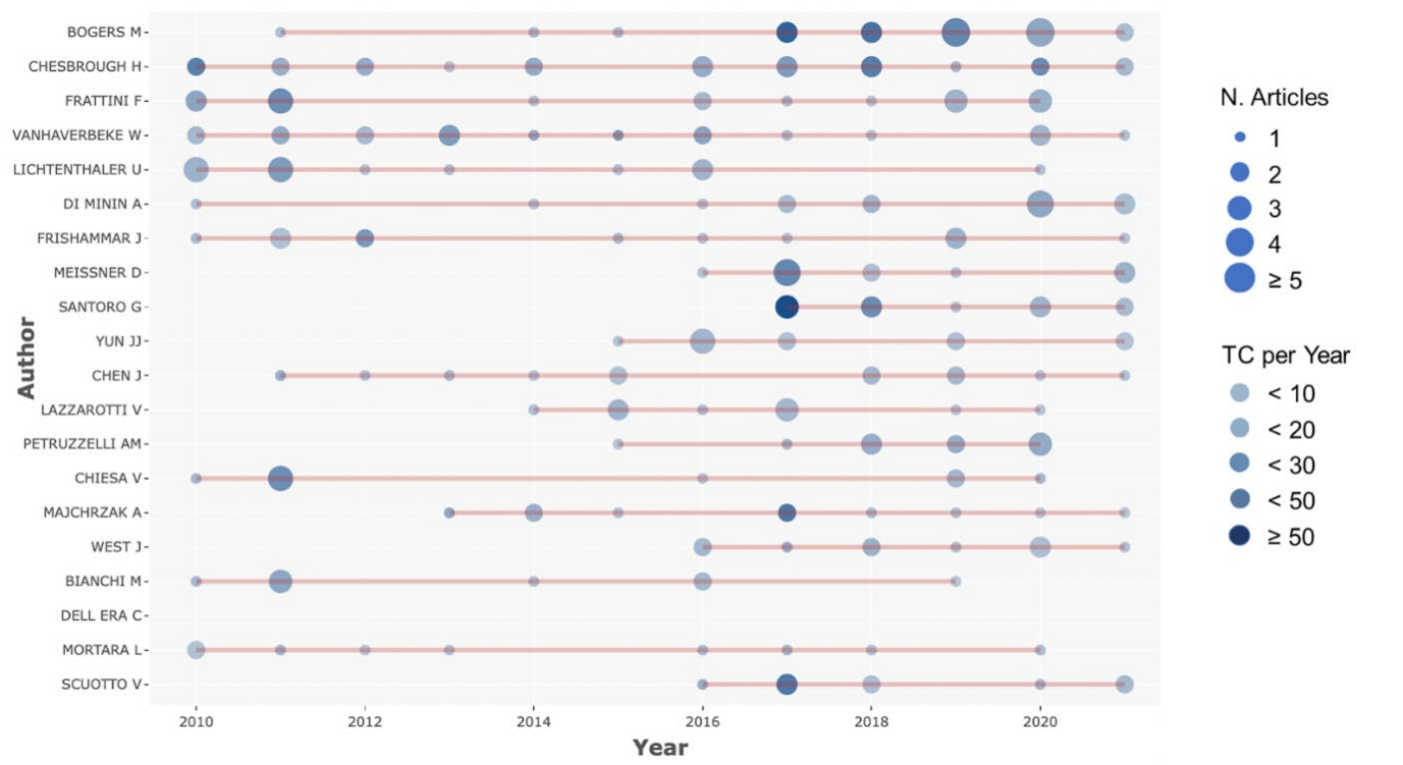
Top authors’ production over time. Source: Biblioshiny, based on the WoS dataset. Note that the bigger the circle is, the more articles have been published by the author in that year. The darker the circle is the more citations have been received per year.

Tables 1 and 2 have introduced the most cited articles (reflecting their impact), while Tables 3 and 4 have introduced the most productive journals (indicating their productivity). Impact and productivity are two of the most relevant aspects in academia. In Fig. 3 we combine these two metrics to outline authors’ relevance within the open innovation field. Therefore, we provide an overview of the top 20 most productive and cited authors from 2010 to 2021. The productivity was evaluated through the number of articles published by these authors in the given period of time. Conversely, the impact was evaluated by considering the number of citations received each year, as the legend in Fig. 3 explains. The figure shows how some authors have constantly contributed over the years in terms of the numbers of both publications and citations. It is not surprising that Chesbrough, who coined the term ‘open innovation’ in 2003 (Chesbrough 2003) has made a constant and linear contribution to the field over the years. This contribution is even underestimated in this graph, since Chesbrough often publishes books and chapters about open innovation (see, for instance, Chesbrough 2003; Chesbrough et al. 2006; Chesbrough et al. 2014; Chesbrough 2019). These books and chapters have a great impact on scholars and practitioners alike, but they are not included in this bibliometric analysis, which focuses only on peer-reviewed articles. Other scholars, such as Frattini and Vanhaverbeke, have published intensively about open innovation for the whole time period, while others, such as Frishammar and Mortara, have published constantly but less intensively. Other scholars, such as Bogers and Di Minin, have impressively increased both their productivity and impact over the last years. Finally, new scholars have emerged in the open innovation research landscape in the time period 2016‒2021, such as Santoro and Scuotto, whose favourite arguments principally cover the topics of knowledge management and digital transformation.

## Open innovation knowledge structure

In this section, we address the second research question of this study:

What are the different knowledge clusters that emerge from open innovation literature?

**Fig. 4:**
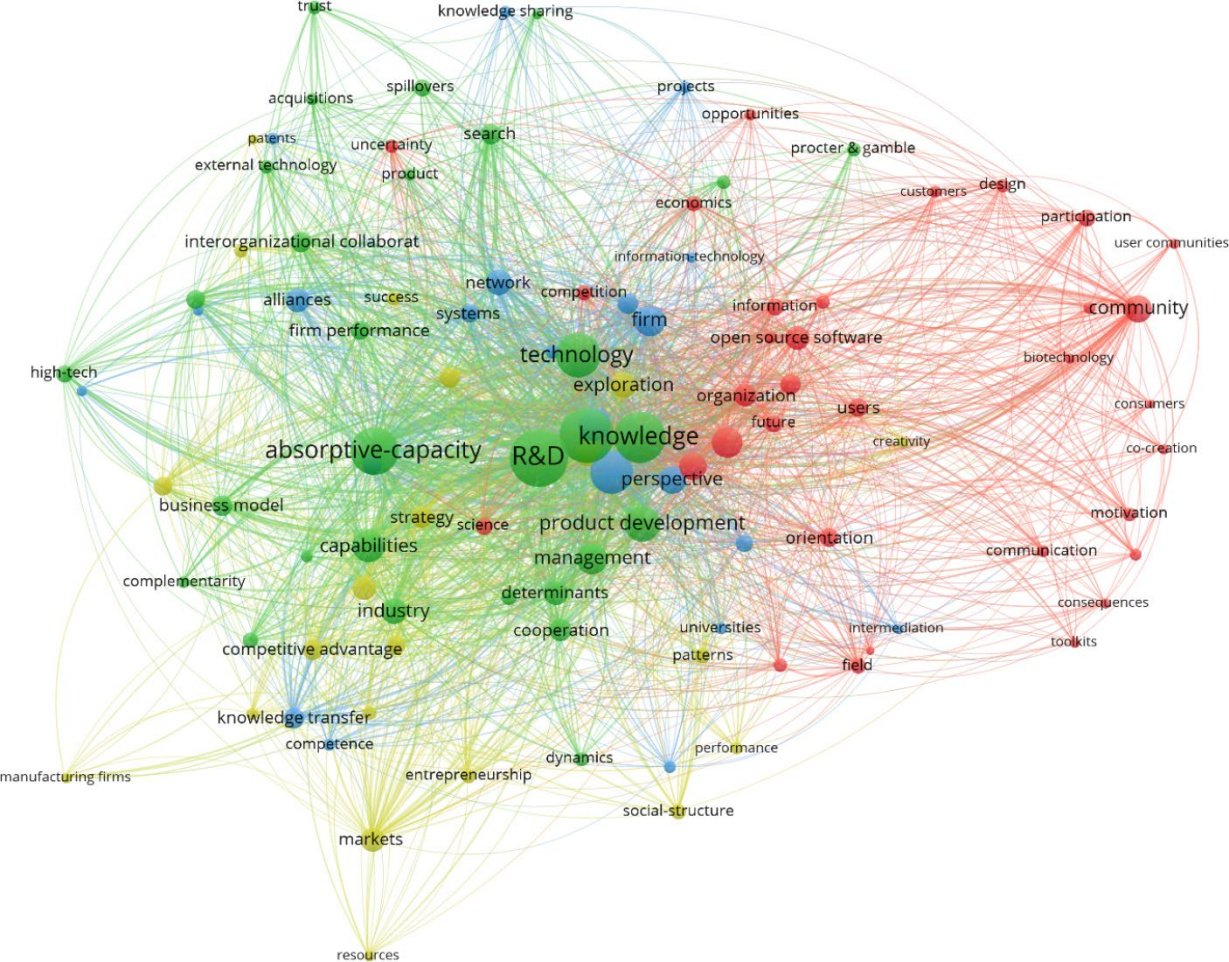
Keyword co-occurrence analysis for the time period 2010‒2015. Source: Authors’ elaboration in VOSviewer software

**Fig. 5:**
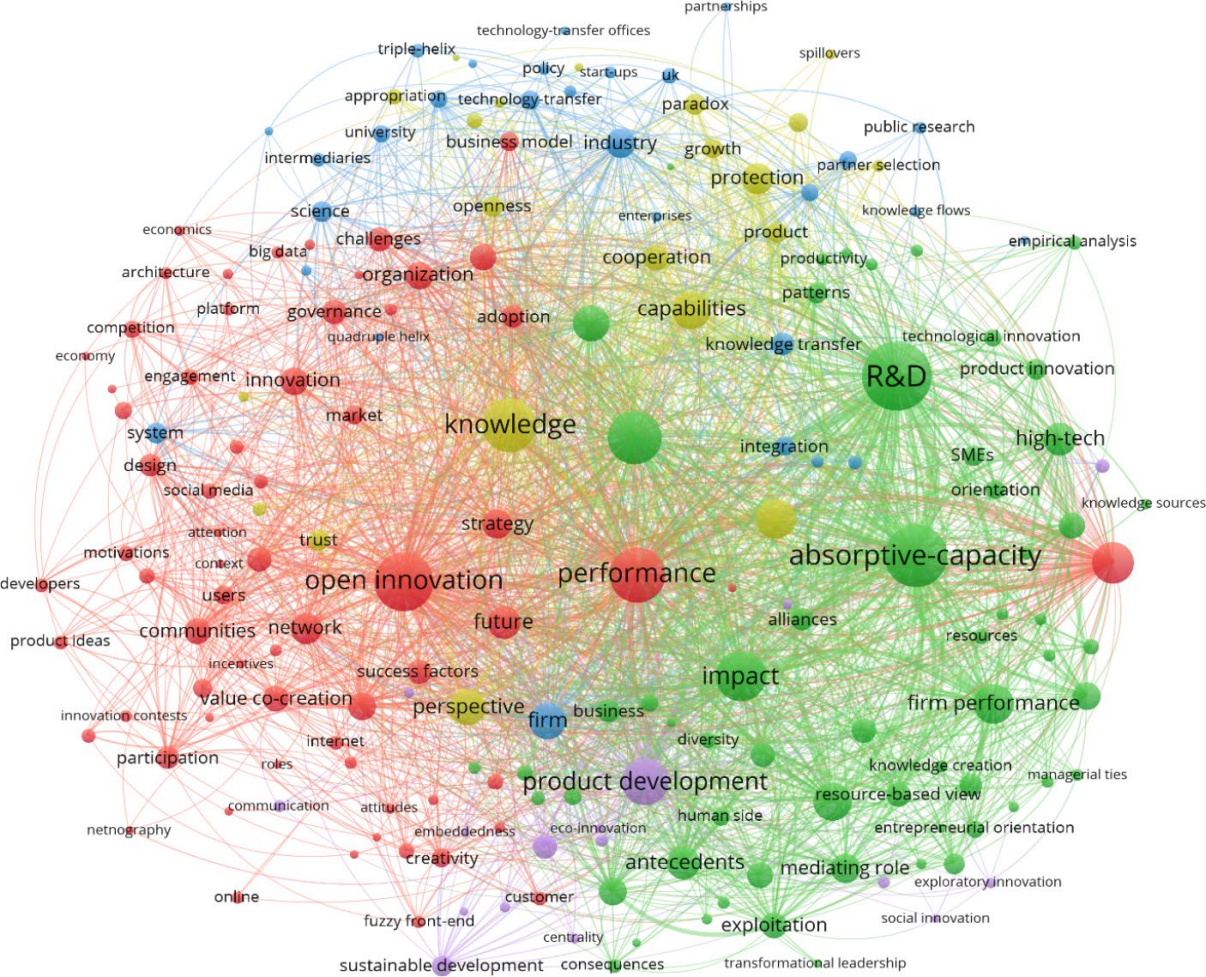
Keyword co-occurrence analysis for the time period 2016‒2021. Source: Authors’ elaboration in VOSviewer software.

The main results from the analysis of the open innovation knowledge structure are summarized in Figs. 4 and 5, which illustrate the clusters resulting from the keyword co-occurrence analysis on VOSviewer. More precisely, we employed network analysis techniques to investigate the connections occurring between similar concepts. In particular, the more co-occurrences were identified, the more the node (i.e., a specific keyword) is central in the network. The more each couple of keywords was used by scholars at the same time, the closer and more robust is the link between them. The higher the occurrences of a keyword are, the bigger is its node (De Bernardi et al. 2021). Only keywords with a minimum cluster frequency of 5 were considered. For a more coherent visual presentation, only the most influential keywords are displayed in the figures (Manesh et al. 2020). An illustrative set of keywords is also presented in Tables 5 and 6. The analysis has been conducted by considering 2010‒2015 and 2016‒2021 as two different time periods.

**Table 5:** Exemplary keyword list of the four clusters characterizing the knowledge structure of scientific literature about open innovation in the time period 2010‒2015

Red cluster Sub-topics: User innovation and value co-creation	Green cluster Sub-topics: Inbound open innovation	Blue cluster Sub-topics: Knowledge management and open innovation governance	Yellow cluster Sub-topics: Open innovation strategy
Co-creation	Absorptive capacity	Alliances	Competitive advantage
Community	Capabilities	Information technology	Dynamic capabilities
Creativity	Cooperation	Intellectual property	Exploitation
Customer	External technology	Knowledge sharing	Exploration
Design	Knowledge	Knowledge transfer	Manufacturing firms
Motivation	Product development	Network	Market orientation
Open source software	R&D	Open innovation	Markets
Organization	Search	Project	Reesource-based view
Participation	Spillover	Systems	Resources
Users	Trust	Universities	Strategy

Source: Authors’ own elaboration.

**Table 6:** Exemplary keyword list of the five clusters characterizing the knowledge structure of scientific literature about open innovation in the time period 2016‒2021

Red cluster Sub-topics: Opportunities and challenges	Green cluster Sub-topics: Human side of open innovation	Blue cluster Sub-topics: University-industry-government collaboration	Yellow cluster Sub-topics: Tensions and paradoxes in open innovation	Purple cluster Sub-topics: Open innovation and sustainability
Challenges	Absorptive capacity	Academic entrepreneurship	Appropriation	Communication
Future	Alliances	Ecosystems	Capabilities	Competence
Information systems	Dynamic capabilities	Industry	Cooperation	Eco-innovation
Markets	Human side	Knowledge spillovers	Coopetition	Embeddedness
Open innovation	Impact	Knowledge transfer	Intellectual property	Environmental innovation
Performance	Microfoundations	Policy	Knowledge	Interorganizational collaboration
Platform	Product innovation	Public research	Openness	Product development
Strategy	R&D	Start-ups	Paradox	Research agenda
Users	Resource-based view	Triple helix	Perspective	Social innovation
Value co-creation	SMEs	Universities	Protection	Sustainable development

Source: Authors’ own elaboration

### Knowledge structure: time period 2010‒2015

The analysis shows four different clusters in the time period 2010‒2015 (Fig. 4). The red cluster (33 items) is more focused on co-creation processes and the figure of users as innovators. The green cluster (30 items) mainly refers to the inbound model of open innovation. The blue cluster (22 items) is focused on knowledge management and governance issues. Finally, the yellow cluster (21 items) includes keywords related to the strategic aspects of open innovation.

#### Red cluster: user innovation and value co-creation

The open model of innovation challenges the underlying precept of the vertically integrated model, according to which innovation must rely on in-house R&D (Chesbrough et al. 2003). On the contrary, it advances the idea that organizations are much more competitive when they co-create innovation through a distributed process across multiple stakeholders in a value network (Bogers and West 2012). One stakeholder group that has received greater attention is precisely that of individual users. The first cluster mainly includes several keywords referring to the role of individuals (e.g., users, lead users, customers, and consumers) as knowledge providers in the innovation process. Although open and user innovation have different fathers (i.e., Chesbrough and von Hippel, respectively), theoretical underpinnings, and assumptions, they both reject the traditional idea that innovation must be created and commercialized within a single organization (Piller and West 2014). Indeed, individual users, consumers, clients or customers represent one of the most significant sources of open innovation that firms can draw upon (von Hippel, de Jong, & Flowers 2012). They can make effective use of their experience to communicate their needs and preferences (Bogers et al. 2010). As noted more recently by Bogers et al. (2017), such users can be motivated to co-produce innovations by collaborating with firms for several reasons such as benefit from using the solution, enjoying the process of doing so, or gaining symbolic capital in the form of peer recognition. Individual users may contribute in several ways to co-create value with firms. Marketing studies, for instance, have highlighted how the role of consumers is no longer exclusively relegated to consume products and services but also to produce them, in a dialectical endeavor that drives firms to a more accurate identification of consumers’ needs (Lusch and Vargo 2014). User communities often bring together people with shared interests to collaboratively build something, which can be shared with anyone inside or outside of the community, as in the case of open source communities. Open-source software communities, for instance, refer to a new paradigm of software creation and development based on hundreds or even thousands of developers and users organized in the form of a virtual community (Iskoujina and Roberts 2015; Martínez-Torres and Díaz-Fernández 2014). Indeed, this distributed production process would not be possible without bringing together different knowledge bases distributed across the world and beyond organizational boundaries (Colombo et al. 2014; Perr et al. 2010).

#### Green cluster: inbound open innovation

We have identified as an umbrella term of this cluster the concept of inbound open innovation. As mentioned in the introduction, different practices fall under the domain of open innovation. However, these various practices have been systematized under three main open innovation processes, namely inbound open innovation, outbound open innovation, and the coupled mode of open innovation (Bigliardi and Galati 2016).

The inbound open innovation mode refers to the process of obtaining innovations from external sources, including search, sourcing, enabling, incentivizing, and contracting (West and Bogers 2014). Firms that perform inbound open innovation have the opportunity to benefit from new ideas and combinations of knowledge, to explore new market opportunities, and to renew their pool of capabilities (Hung and Chou 2013; Martínez Conesa et al. 2017). However, to effectively increase their performance (this cluster includes keywords such as growth and product development that can be considered as possible outcomes of open innovation activities), firms need to be able to utilize external searched knowledge by recognizing its value, integrating it, and applying it to commercial ends. In other words, the theoretical foundations of inbound open innovation build upon the well-established concept of absorptive capacity (Spithoven et al. 2010), defined by Cohen and Levinthal (1990) as ‘the ability of a firm to recognize the value of new, external information, assimilate it, and apply it to commercial ends’ (p. 128). The assumption that absorptive capacity helps firms capitalize on external sources of innovations has opened the doors to two possible hypotheses: (1) higher absorptive capacity is more likely associated with the use of innovations from external sources, or (2) firms with high absorptive capacity will be more successful in such use (West and Bogers 2014). Studies addressing these two macro-hypotheses have reported conflicting insights. De Faria, Lima, and Santos (2010) have suggested that absorptive capacity increases the likelihood that firms will collaborate, while Barge-Gil (2010) has argued that absorptive capacity reduces the need for collaboration. On the other hand, the literature seems to converge around the idea that absorptive capacity increases the chances that remote collaboration will be more effective (de Jong and Freel 2010), and firms with a broader knowledge base are more likely to source external “distant” technologies (Laursen et al. 2010).

Nevertheless, although the concept of absorptive capacity has received much attention in open innovation studies, the role of other organizational capabilities in support of open innovation practices is still overlooked. As an early attempt to complement the absorptive capacity concept through a more integrated view of the capabilities needed to conduct open innovation successfully, Lichtenthaler & Lichtenthaler (2009) have introduced a capability-based framework that considers not only inward but also outward knowledge transfer. Only recently, however, scholars have started focusing on the other two modes of open innovation (i.e., outbound and coupled) to explore new sets of skills and capabilities (e.g., Bertello et al. 2022b; Cheah & Yuen-Ping 2021; Ungureanu et al. 2020).

#### Blue cluster: knowledge management and open innovation governance

As aforementioned, the open innovation model refers by definition to a ‘distributed innovation process based on purposively managed knowledge flows across organizational boundaries’ (Chesbrough and Bogers 2014, p. 17). Organizations have indeed increasingly recognized the importance of working with external partners through partnerships that facilitate access to knowledge in order to innovate in fast-changing environments. Therefore, in order to streamline their journey towards open innovation, firms must adopt knowledge management systems that are able to foster the diffusion, sharing and transfer of knowledge within and across firms (Chiaroni et al. 2011). Effective knowledge management systems are associated with (1) the use of technological platforms and information and communication technology (ICT) tools, and (2) the adoption of appropriate intellectual property management systems preventing the opportunistic behaviors of partners (ibid.).

Many authors have highlighted the role of ICT in supporting the shift towards open innovation practices. For instance, Cui et al. (2015) found that the alignment between information technology flexibility and breadth enhances innovation radicalness and innovation volume, whereas the alignment between information technology integration and depth positively affects innovation volume only. In general, open innovation literature has given a great boost to knowledge management research to consider not only internal but also external knowledge management processes.

Knowledge sharing and transfer processes are not only facilitated by the development of ITC tools but also by the introduction of intellectual property rights. Moreover, although open innovation suggests that knowledge must not be protected but rather shared, firms often need to protect their intellectual assets and exclude others from using their own ideas and inventions. The management of intellectual property rights (IPRs) in firm-to-firm collaboration determines the value appropriation potential for the network partners and positively influences the success of open innovation projects (Leten et al. 2013), especially in networks and alliances that form around projects whose nature is temporary and social capital not yet consolidated. The governance of open innovation projects therefore becomes of the utmost importance to make inter-organizational collaboration more effective. Hagedoorn and Zobel (2015), for instance, have found that firms active in open innovation have a very strong preference for the governance of their open innovation relationships with other firms through formal contracts. Also, despite the open nature of open innovation, firms still see IPRs as highly relevant to the protection of their innovative capabilities. Furthermore, Bogers (2011) has shown how firms cope with the tension between knowledge sharing and protection, highlighting the role of an open knowledge exchange strategy and a layered collaboration scheme with inner and outer members, proposing licensing as a tool to further implement such coping strategies. Despite the importance of IPR management, other governance strategies are nevertheless necessary to orchestrate actors across a network. The temporary nature and the presence of multiple, often unknown, partners with different expectations and goals make it difficult to orchestrate actors across a network. Intermediation can thus make collaboration more effective by means of boundary spanners and knowledge brokers that connect the range of different organizations and knowledge needed to create successful innovation (Gassmann et al. 2010; Sieg et al. 2010).

#### Yellow cluster: open innovation strategy

Research on open innovation has been consistently advanced by strategy and innovation scholars. This cluster includes several terms that are key for the strategic management of open innovation. Terms such as markets, market orientation, and financial performance highlight how firms engaging in open innovation activities are substantively driven by market logic and financial concerns. While strategic scholars have insistently argued that the competitive advantage of an organization relies on its internal resources (Barney 1991), the open innovation model highlights how these resources can be co-created and co-regenerated by interacting with a wide network of stakeholders (West and Bogers 2014). Open innovation often represents the best innovation model to achieve competitive advantage in a world often characterized by hostile environmental conditions and challenging trends such as digitalization and globalization, since organizations do not have enough internal resources (in terms of skills, competences, time, financial resources, etc.) to turn these challenges into opportunities and to achieve a sustained competitive advantage (Igartua et al. 2010). The resource- based view has thus been reconceptualized over the years and new related concepts have emerged that try to provide a response on how organizations can successfully sustain their competitive advantage in the face of the increasing volatility of business environments that impose continuous technological and market changes. A concept that has been used with constancy by open innovation scholars is that of dynamic capabilities (Bogers et al. 2019). Defined by Teece, Pisano, and Shuen (1997) as the ‘firm’s ability to integrate, build, and reconfigure internal and external competencies to address rapidly changing environments’, dynamic capabilities provide a theoretical lens to complement the resource-based view, as we will discuss further in Sect. 5. As highlighted by Vanhaverbeke and Cloodt (2014), scarce, unique, and difficult-to-imitate resources can turn into competence traps as firms get stuck with resources that become rapidly irrelevant in the face of sudden technology and business environment change. Hence, sustaining a competitive advantage goes beyond the ownership of difficult-to-imitate resources and demands difficult-to-replicate dynamic capabilities (Teece 2007).

### Knowledge structure: time period 2016‒2021

The analysis shows five different clusters in the time period 2016‒2021 (Fig. 5). The red cluster (33 items) outlines relevant opportunities and challenges in open innovation research. The green cluster (30 items) focuses on the human side of open innovation. Terms from the blue cluster (29 items) mainly relate to university-industry-government collaboration. The yellow cluster (21 items) refers to tensions and paradoxes in open innovation research. Finally, the purple cluster (16 items) introduces terms at the intersection between sustainability and open innovation.

#### Red cluster: opportunities and challenges

We have labeled this cluster ‘opportunities and challenges’. Over the last years, new tools and new trends have contributed to promoting new ways of organizing open innovation. These tools and trends are bearers of both opportunities and challenges. Connectivity and collaboration among actors is enabled by emerging models based on the use of digital platforms (Nambisan et al. 2018) and emerging technologies such as the Internet of Things and big data analytics to implement information flows (Scuotto et al. 2016, 2017). Digital platforms, for instance, create a digital space where firms can share resources, organize interactions, and guide users and customers towards value (co)creation (Gawer and Cusumano 2014; Parker, Van Alstyne, & Choudary 2016). Decentralized and distributed innovation is also benefitting from the development of increasingly advanced information systems that support data-intensive applications to enhance the efficacy of service delivery to citizens and foster idea generation from a myriad of knowledge sources, which could be geographically dispersed (Kankanhalli et al. 2017). After an initial phase, where open innovation was deliberately promoted by frontline practitioners, principally large and/or high-tech firms, to improve technical efficiency by utilizing external innovations internally, and commercializing internal innovations externally (Bertello et al. 2022a), open innovation principles are now increasingly adopted by governments and the public sector (Ham et al. 2015). On the one hand, governments are setting up funding schemes based on subsidies and incentives to attract organizations of different natures around open innovation projects (Bertello et al. 2022a). On the other hand, public sector organizations are increasingly recognizing the value of involving citizens and users in the shaping of public services (Forliano et al. 2020). Although the public sector is at the early stages of the adoption of open innovation (Ham et al. 2015), innovation contests such as hackathons and more generally, crowdsourcing initiatives, are gradually becoming established open innovation tools (Cricelli et al. 2022), especially to solve wicked problems (Almirall et al. 2014; Brunswicker et al. 2017). During the COVID-19 pandemic this phenomenon has intensified further due to the social distancing imposed by governments and the need to mobilize the crowd to tackle complex and evaluative issues (De Bernardi et al. 2021; Vermicelli et al. 2021). Many contests have spread through the internet to fight the virus, opening the doors for multi-actor collaborations and collective problem solving, often driven by intrinsic motivations. Although trends such as a greater variety of information, incentives to open innovation, and increased democratization of innovation processes can be easily linked to unprecedented opportunities for open innovation as a field of studies, some scholars have recently invited other researchers to explore the challenges behind the opportunities (Stanko et al. 2017). First, an increasing amount of information also implies that both private and public organizations ensure that the data sets released are technically accurate as well as interpretable, and that privacy requirements are satisfied (Kankanhalli et al. 2017). Second, incentives to open innovation may also attract opportunistic behaviors (Wang et al. 2021) as well as social-conditioned organizational responses to public policies that may result in decoupling policy from practice (Bertello et al. 2022a). Third, although online calls for citizen participation seem to invoke more inclusive models of innovation, they also potentially (re)produce new patterns of exclusion in relation to technology (e.g., exclusion of those who are not tech-savvy enough), the model of competition (e.g., in selective innovation contests, who decides or judges may often result in biases), and site of interaction (e.g., lack of access to fast broadband internet and suitable devices) (Mair and Gegenhuber 2021).

#### Green cluster: human side of open innovation

This cluster reflects the increasing attention towards the human side, and more specifically, the microfoundations of open innovation to understand how individuals’ characteristics aggregate at the organizational level (Felin et al. 2015).

Despite being at its early stages, the study of the microfoundations of open innovation is rather variegated in terms of theoretical underpinnings and methodological tools. Generically, the notion of microfoundations of open innovation resonates well with the resource-based view (Bertello et al. 2022b). The list of strategic resources which are found to promote competitive advantage in firms includes, among others, human resources (Pereira and Bamel 2021). The microfoundational approach has thus been principally used to reveal how individual characteristics aggregate at the organizational level to explain organizational capabilities such as absorptive capacity, dynamic capabilities, and relational capabilities that are expected to lead to higher open innovation performance. In this regard, Distel’s study of 106 medical technology firms has revealed that knowledge workers’ cognitive process of perspective taking and their creative behavior are important microfoundations of absorptive capacity (Distel 2019; Bertello et al. 2022b) have instead identified openness to others, assertiveness, and balancing skills as key individual determinants for enabling organizational effective use of internal and external knowledge. Research on the microfoundations of open innovation has also explored the relationship between CEO characteristics and open innovation modes (Ahn et al. 2017) and between knowledge diversity of the firm’s employees and firm-level openness (Bogers et al. 2018b). While Ahn et al. (2017) have drawn on the upper echelons theory, which suggests that organizational outcomes are partially predicted by managerial background characteristics of the top level management team (Hambrick and Mason 1984; Bogers et al. 2018b), similarly to other studies (e.g., Albats et al. 2020), have combined human capital, creativity, and learning theories to explore the human side of open innovation. Other studies have qualitatively developed more comprehensive frameworks of the microfoundations of open innovation by identifying leadership skills and managerial competencies (Podmetina et al. 2018) and entrepreneur, employees, and firm level factors that are expected to drive successful open innovation (Santoro et al. 2020).

#### Blue cluster: university-industry-government collaboration

Innovation studies have increasingly paid attention to the interactions occurring among organizations on a geographical scale that ranges from local to supranational levels (Lee et al. 2020; Vlaisavljevic et al. 2020), gaining insights from system, and, more recently, ecosystem perspectives (e.g., Radziwon & Bogers et al. 2019). Traditionally, the innovation literature has emphasized the importance of public intervention in boosting R&D and innovative performance (Bellucci et al. 2019). Recently, policy makers have increasingly adopted funding schemes that incentivize collaboration between different actors such as universities, firms, and governments. Governments’ efforts to promote collaborative innovation with specific policies and funding schemes (Jugend et al. 2020) indeed go hand in hand with universities’ enhanced role in innovation and technology transfer commercialization of research (Schmitz et al. 2017) as well as firms’ intention to move from in-house research and development to collaboration with complementary partners (Radziwon and Bogers 2018). Most of the terms included in this cluster (technology-transfer, technology-transfer offices, knowledge transfer, academic entrepreneurship, universities, and industry) are typically used in the “triple helix” literature (see Etzkowitz & Leydesdorff 2000; Etzkowitz & Zhou 2017), a stream that explores collaborative dynamics alike, but whose implications differ from open innovation research in that more emphasis is placed on policy and regional development rather than firms’ competitive advantage (Leydesdorff & Fritsch 2006).

Recently, however, also open innovation scholars have started exploring how policies influence open innovation and vice versa, producing controversial insights. While some studies have highlighted the benefits for firms responding to publicly sponsored R&D collaboration (e.g., Bogers et al. 2018a; Oguguo et al. 2020), others highlighted how open innovation policies may also have unintended consequences such as ceremonial adoption or disengagement (e.g., Bertello et al. 2021a; Bertello et al. 2022a), thus highlighting the need for more sophisticated policy intervention to effectively incentivize collaborative behaviors in firms (see Ahn et al. 2020; Marullo et al. 2020). Despite the potential complementarity between universities and industry, several questions remain unanswered, such as how to manage intellectual property rights to mitigate knowledge spillovers and how to manage multiple, potentially conflicting, institutional logics (Lafuente and Berbegal-Mirabent 2019; Holgersson and Aaboen 2019). This cluster also counts another term, quadruple helix, which extends the concept of the triple helix. As aforementioned, citizens are assuming an ever-increasing role in open innovation practices. Indeed, the concept of a quadruple helix highlights the need to consider civil society as a further, valuable source of knowledge to co-create the future together with academia, industry, and governments and to drive structural changes that go beyond the scope of any single organization in order to enhance sustainability development at a systemic level (Carayannis et al. 2018; Del Giudice et al. 2017). The literature seems to suggest a convergence between these two streams. Originally, the triple helix literature has developed separately from the open innovation literature, with different leading scholars representing the two research streams, respectively. This is also confirmed by the fact that the word triple helix is not present in the keyword co-occurrence analysis in the timeframe 2010‒2015. However, differently from the previous time period, the most cited publications drawing on the concept of the quadruple helix from 2016 to 2021 (e.g., Miller et al. 2018; Del Giudice et al. 2017) are authored by scholars who are also active in the open innovation community.

#### Yellow cluster: tensions and paradoxes in open innovation

In the previous paragraphs, it has already emerged that open innovation can be rife with tensions that emerge when different organizations claim intellectual property rights, as well as when they respond to different institutional or organizational logics. One approach that is gaining increasing attention in the field of open innovation studies to explore how organizations deal with tensions is the paradox lens (Lauritzen and Karafyllia 2019). Management and organization literature has defined the paradox as a ‘persistent contradiction between interdependent elements’ (Schad et al. 2016). A paradox thus includes three elements. First, the elements that are part of the tension are contradictory. Second, they appear interdependent and as such equally important to address. Third, the tensions arising from their opposing, yet interdependent nature persist over time. Smith and Lewis (2011) have identified four categories of paradox (i.e., learning, belonging, organizing, and performing). Following this typology, the two categories of paradoxes that have been addressed more by open innovation studies are learning and organizing paradoxes. Paradoxes of learning surface as dynamic systems change, renew, and innovate. Paradoxes of organizing, instead, become salient when complex systems create competing designs and processes to achieve a desired outcome (ibid.). The tension between change and stability represents a typical example of a learning paradox. In this regard, the tension between knowledge exploration and knowledge exploitation (rooted in ambidexterity literature) has attracted the attention of several scholars (e.g., Bresciani et al. 2018; Vrontis et al. 2017), although it has not always been explored from the perspective of paradox theory. The most recurrent tensions in open innovation research refer, however, to paradoxes of organizing and, more specifically, the tensions between cooperation and competition, for which the term coopetition has been coined (Bouncken et al. 2015; Roig-Tierno et al. 2018), and the tensions between openness and protection (Foege et al. 2019). This is probably the tension that has attracted most attention among open innovation scholars, so much so that it has often been labeled as the open innovation paradox by definition (Bogers 2011; Laursen and Salter 2014). In the years to come, the ongoing processes of digitalization and sustainability transformation, and the conditions of change and resource scarcity created by them, are expected to generate and/or make latent new paradoxes (Smith and Lewis 2011) as well as suggest new ways of navigating these tensions in open innovation contexts.

#### Purple cluster: open innovation and sustainability

We live in a world that is increasingly characterized by complex problems with far-reaching societal implications, such as climate change, poverty, energy and water supply, natural disasters, and pandemics, recently conceptualized as ‘grand challenges’ by the management and organization literature (George et al. 2016). Grand challenges transcend the interests or influence of individual organizations or local communities and require collective, coordinated, and sustained efforts from multiple different actors (Ahn et al. 2019; Ricciardi et al. 2021). In this regard, the open innovation community has the huge opportunity to offer possible new solutions to grand challenges on the one hand, while taking lessons on how to move forward the concept of open innovation on the other. The purple cluster groups together some concepts that try to link open innovation with sustainability (e.g., eco-innovation, environmental innovation, social innovation), and others, such as sustainable development, that fall under the wide umbrella of sustainability research. The concept of open eco-innovation emerged in the mainstream literature in 2015, after Ghisetti et al.’s (2015) publication in Research Policy. In their work, the authors extended the open innovation paradigm to environmental innovation, laying the foundations for a research stream that has attracted the attention of scholars at the intersection between management, engineering and environmental studies. This cluster also includes the keyword social innovation. A consistent part of the literature on social innovation has focused on social innovators as individual heroes who can leverage unusual creativity, charisma, and foresight to identify opportunities where others see problems (van Wijk et al. 2019). However, the interdependencies among the multiple systems and actors that characterize social problems have called for research that investigates social innovation as the result of collective and dynamic actions (Phillips et al. 2015). This is in line with the three goals that social innovation should address according to Murray et al. (2010): creating new ideas (product, services, and models), meeting social needs, and creating new social relationships and collaborations. Given these premises, Chesbrough and Di Minin tried to introduce the concept of open social innovation in 2014 (Chesbrough and Di Minin 2014). After an initial period of stasis, this concept has been brought to the fore by many scholars who have explored how open social innovation is implemented in different empirical contexts such as fab labs (e.g., Rayna and Striukova 2019), hackathons (e.g., Mair and Gegenhuber 2021), and crowdsourcing (e.g., Chandra et al. 2021; Randhawa et al. 2019). Social embeddedness, another concept emerging from our analysis, can be easily linked to social innovation. Research on embeddedness has a long tradition in entrepreneurship research, dating back to the Austrian school and particularly Lachmann’s (1976) research. Embeddedness allows entrepreneurs to become part of the local structure and to access as well as constitute both latent and readily accessible resources and opportunities (Jack and Anderson 2002). When social entrepreneurship (or social innovation) is at stake, an entrepreneur strongly embedded in a local context can act as a catalyst of social learning (Cantino et al. 2017; Seelos et al. 2011), identifying community needs more easily and strengthening social relationships.

Although these streams of studies are expected to grow rapidly, research about open innovation and its role in tackling sustainability issues and grand challenges is still marginal, as Fig. 5 shows. As highlighted by Kimpimaki et al. (2022), despite the recent emergence of concepts that try to connect openness to sustainability, such as ‘sustainable open innovation’ (Bogers et al. 2020), ‘open sustainable innovation’ (Cappa et al. 2016) and ‘open social innovation’ (Chesbrough and Di Minin 2014), a broader and more general understanding of this area of research remains underdeveloped.

## Network analysis of the theoretical landscape

The concept of open innovation has been primarily developed by observing changing innovation management practices in companies (Chesbrough 2003, 2006), thus following a practice-based approach that explains to some extent why research on open innovation has not been grounded systematically in prior management research. In order to develop a better theoretical grounding of open innovation, Vanhaverbeke & Cloodt (2014) have highlighted the need to link open innovation to the strategy literature and to different theories of the firm, such as the resource-based view, relational view, transaction cost economics, resource dependence theory, and real options theory. Moreover, they argued that existing management theories should be combined, as none of them can fully explain how companies benefit from open innovation. Seven years after that article, open innovation still has no solid theoretical foundations. Indeed, scholars are increasingly highlighting the need for a more comprehensive understanding of open innovation by calling for approaches that draw on other literature streams, thus advancing theoretical concepts that might improve the success rates of open innovation collaborations (Randhawa et al. 2016; Stanko et al. 2017). Based on this premise, we selected the 15 most cited empirical articles in the time periods 2010‒2015 and 2016‒2021, respectively. We analyzed these articles carefully to understand the theoretical views or approaches advocated in the studies. In a few cases, especially in the first cluster, a few articles were not explicitly advocating any theory. In some cases, we found that the article under analysis advocated a single theory or approach. In other cases, we found that the article under analysis advocated the joint use of two, or even more, complementary theories or approaches. This allowed us to conduct a network analysis to gain a synthetic overview of which theories and approaches are advocated by the most influential open innovation articles. In this way, it was possible to answer the third research question of this study:

What is the theoretical landscape that emerges from open innovation literature?

### Network analysis: time period 2010‒2015

**Fig. 6:**
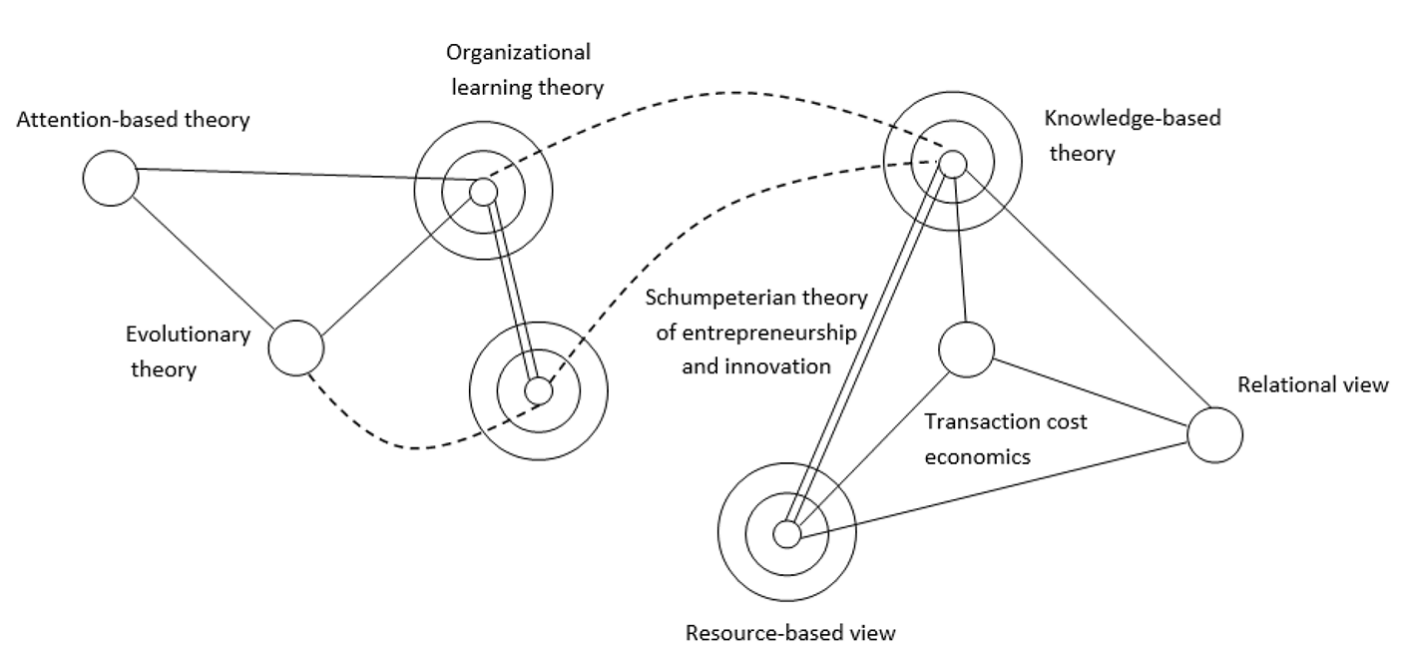
Network analysis of the theories and theoretical approaches advocated in the 15 most cited empirical articles in the field of open innovation for the time period 2010-2015. Source: Authors’ own elaboration. The number of circles corresponds to the number of articles in which a theory or approach is explicitly advocated. The number of lines corresponds to the number of articles in which the two theories have been explicitly linked or co-advocated. Dotted lines represent cross-fertilizations that are not explicitly advocated by the articles under analysis.

The network analysis of the 15 most cited empirical studies in the time period 2010‒2015 (see Table 1 for the full list of papers included) showed the predominance of four theories/theoretical approaches (i.e., organizational learning theories, Schumpeterian theory of entrepreneurship and innovation, resource-based view, and knowledge-based theory). Another four theories (i.e., attention-based theory, evolutionary theory, transaction cost economics, and relational view) are instead mentioned only once. We also identified, although they are not explicitly depicted in the figure, two bridging concepts (dynamic capabilities and absorptive capacity). These two theoretical concepts have significantly informed open innovation research, linking numerous of these theories (see Fig. 6).

Mentioned in 4 out of the 15 most cited empirical studies in the time period 2010‒2015 (Berchicchi 2013; Brunswicker & Vanhaverbeke 2015; Laursen & Salter 2014; Mina et al. 2014), the resource-based view is one of the most influential theories of the firm in the field of strategic management. According to this perspective, a firm requires a unique collection of resources, competencies, and capabilities to be competitive (Barney 1991; Wernerfelt 1984). To create a competitive advantage and capture above-normal rates of returns (i.e. rents), these resources must, by definition be scarce, valuable, difficult-to-imitate, and reasonably durable (Barney 1991; 2001). In other words, it is the result of the use of resources and capabilities that are owned and controlled within the boundaries of a single firm that explains the differences in performance between firms (Bierly and Chakrabarti 1996). This view about how companies develop and sustain a competitive advantage, however, is typically in line with the closed innovation framework. So, why is the resource-based view one of the most advocated theories to study open innovation? A partial reason may be that both the resource-based view and the open innovation model emphasize the importance of resources and competencies to achieve sustained competitive advantage (Vanhaverbeke and Cloodt 2014). However, how can a perspective that stresses independence and the crucial role of internal resources and capabilities with the open innovation perspective of exploiting the interdependences between complementary resources be aligned? The existing literature has suggested two ways: first, by combining synergistically the resource-based view with other theories; and second, extending it. A synergic combination can be found in the relational view of the firm. The relational view emphasizes that critical resources can and should also be found outside the firm’s boundaries (Dyer and Singh 1998). Collaborating firms that combine resources in unique ways may realize a competitive advantage over others that compete on the basis of a stand-alone strategy. The relational view identifies complementary resources or capabilities of firms as a potential source of inter-organizational competitive advantage: this is in line with a major premise of open innovation to consider the sourcing of knowledge from external partners a source of competitive advantage (Alexy et al. 2018). The relational view of the firm can therefore also complement the knowledge-based theory. The main assumption behind the knowledge-based view is, in fact, that the most important strategic resource for sustained competitive advantage within organizations in a knowledge-based economy is the knowledge owned by companies.

Attempts to extend the resource-based view and knowledge-based theory can benefit from the theoretical concept of dynamic capabilities. By relying on internal capabilities only, companies are likely to get stuck in “core rigidities” (Leonard-Barton 1992) or the so-called familiarity trap (Ahuja & Lampert 2001), reducing the chances that firms take advantage of new technological opportunities (Lunn and Martin 1986; Levin et al. 1985). Teece has therefore introduced the concept of dynamic capabilities (Teece 2007), referring to firms’ ability to expand their narrow search horizon and combine internal and external knowledge that originates in the core as well as the periphery of their business ecosystem. According to Teece, firms can overcome the limits of internal learning by making use of sensing, seizing, and reconfiguring capabilities. More specifically, sensing capabilities support the discovery and evaluation of valuable external knowledge, seizing capabilities sustain adaptation and integration processes enabled by internal investments in R&D, good governance mechanisms, and the implementation of lateral and vertical communication, while reconfiguring capabilities are particularly relevant to realign the organization to integrate external knowledge and to develop a culture that promotes collaboration (Bogers et al. 2019).

Figure 6 shows how another theory that is particularly recurrent in open innovation works in the time period 2010‒2015 is the Schumpeterian view of entrepreneurship and innovation. Central to our understanding of the innovation process is how organizations search for knowledge (Nelson and Winter 1982). These search processes are described by Schumpeter as characterized by the need for firms to search for and carry out “new combinations” of technologies, knowledge, and markets with the purpose of producing other things, or the same things through different methods (Schumpeter 1982).

In order to aid our understanding of the Schumpeterian view from an open innovation perspective, the concept of dynamic capabilities may be helpful anew. Teece et al. (1997) suggest that dynamic capabilities have the capacity to reconfigure, redirect, transform, shape, and integrate central knowledge, external resources, and strategic and complementary assets. They will allow the firm to respond to the challenges presented by the Schumpeterian ever-changing world, made of competition and imitation (Teece et al. 1997). The Schumpeterian view of entrepreneurship and innovation is linked to the resource-based view but also (dotted line) to the knowledge-based view. According to the Schumpeterian rent creation logic, in fact, knowledge and its recombination can be considered a core intangible asset that contributes to building sustained competitive advantage.

Another important theoretical approach that emerges in our network analysis relates to organizational learning theories. Although organizational learning is often seen as a field of study rather than a theory, we have included it in our network analysis since its use in the field of open innovation draws on learning theories (e.g., Bower & Hilgard 1981; Harlow 1949). Organizational learning can be defined as a change in the organization’s knowledge that occurs as a function of experience (Argote and Miron-Spektor 2011; Fiol and Lyles 1985). The knowledge the organization develops can be explicit or it can be tacit and difficult-to-articulate (Kogut and Zander 1992), and it can also be converted from one type to another (Nonaka and Von Krogh 2009). The knowledge can manifest itself in a variety of ways, including changes in cognitions, routines, and behaviors. Organizational learning is therefore linked to the knowledge-based view. A firm’s sustained competitive advantage depends, in fact, on both the firm’s ability to embed knowledge in a variety of repositories or knowledge reservoirs, including tools, routines, social networks, and transactive memory systems (Argote and Ingram 2000; Walsh and Ungson 1991), but it also resonates well with the open innovation principles, since organizational learning often depends on the combination of internal and external knowledge that originates in the core as well as the periphery of their business ecosystem. Organizational learning is often mediated by the concept of absorptive capacity, another bridging concept for the theories that support a better understanding of open innovation. Absorptive capacity suggests, by definition, that higher levels of absorptive capacity lead to firms’ better understanding of the knowledge received, making it easier to unlock and capture the intrinsic value of such knowledge and apply it for commercial purposes (Carayannis et al. 2012). In the same way as the dynamic capabilities concept, the intrinsic dynamism of the notion of absorptive capacity is appropriate for understanding open innovation in fast-changing settings.

Another perspective that has been used to explore open innovation is the transaction cost theory. Open innovation is about setting up relations with external innovation partners. The inter-organizational relations that occur between firms reflect transactions between different legal entities. Firms can, in fact, collaborate in many different ways with their innovation partners and the choice between these different types of collaboration is at the core of the transaction costs economics (Williamson 1989). Transaction costs tend to increase when fear of opportunism and asset specificity are higher. However, collaboration can potentially cope with a high degree of asset specificity and can also lower uncertainty and opportunistic behavior by monitoring partners’ performance, balancing their contributions, and creating interactions based on mutual reciprocity and long-term relationships (Bogers 2011; Vanhaverbeke and Cloodt 2014), however, have proposed to integrate the transaction cost economics with the transaction value theory, since ‘careful observation of open innovation deals reveals that companies choose a particular type of collaboration governance to jointly maximize the [strategic] value of a transaction rather than to minimize transaction costs’ (Vanhaverbeke and Cloodt 2014, p. 263). Moreover, the link between open innovation and the theories of learning suggests that the value of cooperation in open innovation contexts is based not only on transaction costs objectives but also on learning opportunities (Kogut and Zander 1992). Transaction cost economics and the resource-based view (as well as the knowledge and the relational-based view) are fundamentally different. The former invokes a contractual view whose focus is explicit and implicit contracts between employers, employees, and other contractors. This view, however, has been criticized for neglecting non-contractual relations that affect transactions such as trust and loyalty, and for providing static explanations of organizational arrangements. On the contrary, the resource-based view places organizations in a disequilibrium environment where they develop resources and skills to achieve competitive advantage. Despite their differences, however, resource-based view and transaction cost economies also complement each other (Argyres and Zenger 2007; Mayer and Salomon 2006). In this regard, the concept of dynamic capabilities can bridge transaction cost and non-transaction cost arguments into a more comprehensive theory that can be fruitfully used to reveal the dynamics of open innovation. A focus on comparative static explanations, where one organizational arrangement is deemed to have lower (transaction) costs than another, leads to inadequate treatment or neglect of dynamic aspects of the problem, notably learning, innovation, and technological development. As Teece and Pisano (2003) argued, firms do not only deal with transaction costs but also with many types of arrangements where injecting high-powered (market-like) incentives might well be destructive of the cooperative activity and learning (Hodgson 1998).

Another theory emerging from our analysis is the attention-based view. This theory postulates three core principles: first, decision-makers’ focus of attention has an impact on strategic choices and outcomes; second, this attention is contextually situated; third, this situated attention is socially structured (Ocasio 1997). Although this theory is sparingly used in open innovation research, it can potentially gain more relevance, since the view of humans as boundedly rational and characterized by cognitive limits raises issues on how they may allocate their attention and behave in inter-organizational contexts characterized by information overload, multiple and sometimes conflicting goals, and potential opportunistic behavior (Bertello et al. 2022a; Giusti et al. 2020). An attention-based approach can provide insights in exploring how organizations absorb knowledge and how they balance exploration and exploitation activities (Miller & Martignoni 2015).

Finally, our social network analysis also includes one attempt to extend evolutionary economics. Ghisetti et al. (2015), in their Research Policy paper, investigate the effect of knowledge sourcing and absorptive capacity on firms’ environmental innovation, extending the open innovation paradigm to environmental innovation (i.e., open eco-innovation), and contributing to a growing body of literature that analyzes the potential of evolutionary economics to explain sustainable development and environmental policies.

### Network analysis: time period 2016‒-2021

**Fig. 7:**
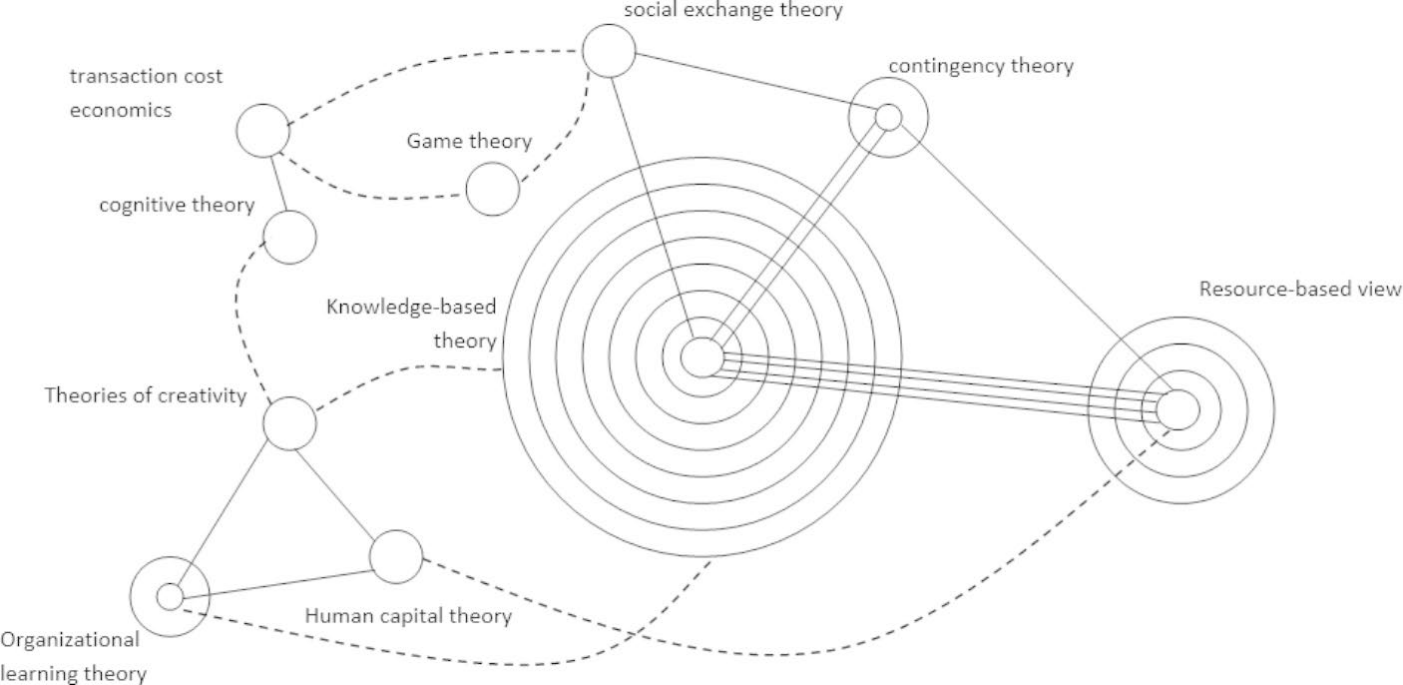
Network analysis of the theories and theoretical approaches advocated in the 15 most cited empirical articles in the field of open innovation for the time period 2016‒2021. Source: Authors’ own elaboration. The number of circles corresponds to the number of articles in which a theory or approach is explicitly advocated. The number of lines corresponds to the number of articles in which the two theories have been explicitly linked or co-advocated. Dotted lines represent cross-fertilizations that are not explicitly advocated by the articles under analysis.

The network analysis of the 15 most cited empirical studies in the time period 2016‒2021 (see Table 2 for the full list of papers included) showed the increased predominance of the resource-based view and knowledge-based theory of the firm (recurring 4 and 8 times, respectively), often used in combination. This result can, in part, be explained by the increasing attention (see Table 4) that the Journal of Knowledge Management, one of the leading journals in the knowledge management field, is devoting to the open innovation discourse. Another two theories (i.e., theories of organizational learning and contingency theory) are advocated twice, while a broader range of theories has been explicitly mentioned once (i.e., transaction cost economics, cognitive theory, theories of creativity, game theory, social exchange theory, human capital theory) (see Fig. 7).

A comparison between the network analysis from the two time periods shows that 10 theories have been advocated in the time period 2016‒2021, against only 8 in 2010‒2015. There was an increase also in the number of articles that have used more theories and/or theoretical approaches contemporaneously, as can be seen from the number of circles and the number of lines (22 vs. 20 and 13 vs. 12, respectively). This might suggest that scholars are gradually trying to combine existing theories to capture the complexity of open innovation processes. It can also be seen how, beside the well-grounded conversation around the resource-based view and knowledge-based theory and their use in open innovation studies, the theoretical discourse has also opened the door to micro-level theories. This reflects the recent turn in open innovation studies towards exploring the human side of open innovation (e.g., Albats et al. 2020; Bogers et al. 2018b) by employing a microfoundational approach as a theoretical lens to understand how the organizational context to which individuals are exposed and their actions and interactions influence organizational capabilities (Felin et al. 2015). Indeed, cognitive theories, theories of creativity, and human capital theories have recently been used to reveal the microfoundations of open innovation-related capabilities such as absorptive capacity, relational capabilities, and, more generally, dynamic capabilities. Instead, a macro-level focus (i.e., open innovation in relation to institutions, policies, socio-ecological systems), at least in the most influential papers in terms of citations, is still lacking,

Transaction cost economics is only mentioned in one paper, as in the previous time period. Inter-organizational relationships, however, do not include only the cost of transacting, but also cognitive and organizational costs (Cassiman and Valentini 2016). The social-exchange theory, for instance, has been increasingly adopted by knowledge management scholars to inform research on knowledge sharing (Liao 2008) and knowledge hiding (Perotti et al. 2021). Its underlying assumption is that people rely on cost-benefit analysis to determine the risks and benefits of each decision and behavior. As a consequence, they will be willing to share knowledge when the (perceived) rewards are higher than the (perceived) costs (Lin and Huang 2010; Tsai and Cheng 2012; Wu and Lee 2017). Since the quality of knowledge flows plays a key role in open innovation (Chesbrough and Bogers 2014), the social exchange theory can provide valuable insights to explore the antecedents of knowledge flow (Martinez-Conesa et al. 2017) at both firm and inter-organizational levels.

One paper in our sample (Parker and Van Alstyne 2018) has also made use of game theory as part of an extensive theory that studies optimal decision-making processes. In their paper, Parker & Van Alstyne (2018) developed a sequential innovation model that explores the optimal levels of openness and of intellectual property duration in a platform ecosystem. Based on the first trade-off they identified, closing the platform increases the sponsor’s ability to charge for access, while opening the platform increases the developer’s ability to build upon it. According to the second trade-off, the longer the third-party developers retain rights to their innovations, the higher the royalties they and the sponsor earn, but the sooner those developers’ rights expire, the sooner their innovations become a public good upon which other developers can build.

Finally, a theoretical approach that has been mentioned twice in the 15 articles analyzed in the time period 2016‒2021 is the contingency theory (Martinez-Conesa et al. 2017; Popa et al. 2017), an organizational theory developed in the second half of the twentieth century around the core argument that there is no best way to organize a corporation, to lead a company, or to make decisions. Instead, the optimal course of action is contingent (dependent) upon the internal and external situation. This theoretical approach has informed prior studies arguing that companies’ innovation strategies are contingent on both internal and external factors (Gibson and Birkinshaw 2004; Jansen et al. 2006). Accordingly, previous open innovation research suggests that firms’ migration toward opening up innovation strategies is also influenced by internal and external contexts, from technological development to institutional pressures (e.g., Bertello et al. 2022a; Huizingh 2011). In this vein, the existing literature has suggested that opening up innovation strategies is more suitable in business environments characterized by globalization, competitive intensity, and market and technological turbulence (Akgün et al. 2019; Huizingh 2011).

## Research agenda

Based on the results of the bibliometric and content analysis, we conclude this article by outlining some avenues for future research, thus addressing the fourth research question of this study:

What are the research opportunities that might advance research on open innovation?

More precisely, we outline a research agenda in relation to how to conceptualize, theorize, and research (methods and analytical techniques) open innovation (see Table 7).

**Table 7:** Open innovation: a research agenda

	Research opportunities	Exemplary research questions
**Conceptualization**	Conceptualizations	How to conceptualize open innovation in contexts in which a many-to-many stakeholder approach is preferable to a firm-centric approach? How to conceptualize and analyze open innovation from a practice- and process-based perspective (e.g., open innovating)?
	Definitions	How to adapt existing open innovation definitions to emerging organizational and societal needs? How to (re)define open innovation to outline the tensions that are inherent to sustainable and digital open innovation models?
	Visualization tools	Which visualization tools can best represent sustainable and digital open innovation models? How to adapt the open innovation visualization models (e.g., open innovation funnel) to recent calls for approaches that invoke a pluralistic and recursive view of innovation as a never-ending process?
**Theorization**	Cross-level theorizing	How to integrate the perspective of traditional theories of the firm (e.g., resource- and knowledge-based view) with micro-level theories (e.g., microfoundations, cognitive theories, behavioral theories) and macro-level theories (e.g., institutional theory, social-ecological systems, theory of the commons)?
	Combining theories	Which management, organization, and strategy theories can be combined to capture the complexity of open innovation practices? And how? Which theories from close domains (e.g., sociology, philosophy, psychology, anthropology) can be used to explore open innovation?
	New theorizing	Will existing theories be enough to explain open innovation in the future or should we focus our efforts on grounding new theorizing?
**Research methods and analytical techniques**	Traditional, well established methods	How can we use traditional quantitative methods (e.g., regressions, structural equation modeling) to analyze the dark sides of open innovation? How can we combine quantitative and qualitative methods to capture more detailed insights from open innovation practices?
	New, emerging methods	Which configurations of factors (e.g., human skills and personality traits, organizational structure, organizational culture, and institutional factors) can be associated with open innovation success and failure? How can asymmetrical techniques, which draw on the ontological assumptions of complexity theory, such as fuzzy-set qualitative comparative analysis, support open innovation research? How to explore open innovation from a systems thinking lens? How can netnography, and, more generally, digital ethnography, inform research on open innovation in virtual settings?

Source: Authors’ own elaboration

### Conceptualizing open innovation

A collective endeavor to reconceptualize open innovation first requires a critical reflection on how to define open innovation. Based on the widely accepted definition provided by Chesbrough and Bogers (2014, p.17), open innovation is currently defined as “a distributed innovation process based on purposively managed knowledge flows across organizational boundaries, using pecuniary and non-pecuniary mechanisms in line with each organization’s business model”. Recently, Bogers et al. (2020) have introduced the concept of sustainable open innovation, integrating the previous definition of open innovation with the definition of sustainable development, as developed by the World Commission for Environment and Development report for the United Nations (WCED, 1987). Sustainable open innovation is therefore now defined by Bogers et al. (2020, p. 1507) as “a distributed innovation process which is based on purposively managed knowledge flows across organizational boundaries, using pecuniary and non-pecuniary mechanisms in line with the organization’s business model, thereby contributing to development that meets the needs of the present without compromising the ability of future generations to meet their own needs”. While their new definition responds to the need to adapt existing open innovation definitions to emerging organizational and societal needs, it raises questions about how to do so. We invite scholars to reflect on the existing definitions of open innovation to understand whether they are still valid in light of the disruptive changes that we have outlined in our paper. More specifically, the open innovation community could question whether integrated definitions such as the one provided by Bogers et al. (2020), which combine the core elements of the traditional definition of open innovation with the definition of sustainable development, reflect a sufficiently critical approach to capture and describe with rigor the phenomenon under analysis. Their definition, for instance, stresses how the outcome of open innovation activities is no longer exclusively a firm’s competitive advantage, but also the sustainability of the system in which firms are embedded (Ricciardi et al. 2021). However, the definition seems to overlook the inherent paradoxes of combining market and sustainability logics and short-term and long-term perspectives. Moreover, the ongoing efforts of researchers, practitioners, and policy makers to promote open innovation at a broader, societal level, as well as emerging technologies, such as digital platforms and the Internet of Things, pave the way to explore not only how traditional organizations adapt their structures, processes, and culture to implement open innovation activities, but also how new open innovation models that are not necessarily structured around a focal firm emerge (see Bertello et al. 2021b; Gegenhuber et al. 2023). To conclude, the open innovation research community initially developed around a limited number of scholars interested in the managerial implications of this emerging innovation paradigm. Nowadays, however, contributions to the open innovation research domain come from an increased number of scholars from different communities. As a consequence, the open innovation concept has been expanded and is being applied to a wide set of contexts. Future endeavors to define open innovation should therefore avoid, at the same time, both concept stretching, which may be caused by a unique all-encompassing definition of open innovation, and redundancy, which may result from the multiplication of definitions. (Re)newed definitions must also go hand in hand with new or (re)newed visualization tools that might support conceptualization work in open innovation studies. Therefore, we also encourage scholars to question and adapt existing open innovation visual frameworks, or to develop new ones (Vanhaverbeke 2013). For instance, is the innovation funnel still valid to align open innovation with recent calls for approaches that invoke a pluralistic and recursive view of innovation as a never-ending and constantly (re)negotiated process in which means and outcomes can be considered as mutually enabling and constituent of one another (see Farjoun et al. 2015).

### Theorizing open innovation

Open innovation has principally developed as a field of studies very closely linked to the community of practitioners. Chesbrough himself, who coined the term, has long years of experience in the industry. Open innovation research has therefore privileged practical application over theoretical explanation. As a consequence, we have no specific open innovation theories (Gassmann and Enkel 2004,) but a wide range of applications of existing theories of the firm (Vanhaverbeke and Cloodt 2014), as confirmed by our studies, which registered a massive use of the resource-based view and the knowledge-based theory of the firm. The analysis of the second time period, however, showed that the microfoundations of open innovation are gaining attention, with scholars increasingly eager to explore not only the technical but also the human side of open innovation, as well as the cross-level nature of open innovation by revealing macro-micro-macro level interactions. Combining these insights from the network analysis of theories with the results of our keyword co-occurrence analysis, which showed us how most of the words referring to the technological and to the human side of open innovation were respectively falling into two different clusters in the time period 2016‒2021, we may conclude that we still lack a relational view of these two domains. In other words, we now have increasing insights about how the diffusion and adoption of new technologies impacts firm performance via open innovation practices. We are also gradually gaining knowledge about how human skills and organization culture influence firm openness. However, we still know little about how the technological and the human are constitutively entangled (see Leonardi 2013) and how this entanglement nurtures open innovation endeavors.

On the other hand, what we are also missing in the open innovation literature are macro-level discourses. In this regard, Bertello et al. (2022a) have recently invoked an institutional view of open innovation to consider firms’ efforts to adapt not only to technical pressures but also to societal expectations. According to their view, “the rapid spread of successful cases of open innovation adoption, and the increasing number of public policies promoting open innovation, may lead firms to address it as a way to conform to a societal mandate, or legitimacy, even though these pressures contradict internal needs for efficiency”. This reflection paves the way for exploring the diffusion of open innovation practices among less proactive organizations, as well as the unintended consequences (also called in the open innovation literature ‘dark sides’) of promoting the opening up of innovation processes.

Another theory that might help to explain theoretically open innovation is the stakeholder theory (Shaikh and Randhawa 2022). By conceptualizing firms as organizations at the center of an ecosystem of actors (i.e., stakeholders) who interact with the organizations and who are carriers of different interests, stakeholder theory invites organizations to rethink what creating value means in a complex society where business, social, and environmental concerns are intertwined (Freeman 1984). Moreover, it also suggests that this multiplicity of views and interests can be better achieved through collaborating directly with the different stakeholders (be they employees, customers, universities, governments, etc.). Indeed, recent perspectives that see stakeholders no longer as recipients but rather as creators (or co-creators) of value (Freudenreich et al. 2020), and multi-stakeholder engagement as enablers of collective action when focal firms engage in shared governance forms rather than hub-and-spoke governance models (Bridoux and Stoelhorst 2020), could inspire further reflections on how to manage innovation across distributed networks of stakeholders. Moreover, a multi-stakeholder view of collaborative innovation links well to other theoretical approaches such as the institutional logics perspective and the paradox lens. The institutional logic perspective, for instance, might suggest how to organize open innovation in terms of goal setting, decision making, roles and tasks, communication style, and monitoring in order to navigate multiple, often conflicting logics. The paradox lens, instead, could contribute to shed light on the tensions that characterize inter-organizational contexts: tensions between conflicting institutional logics, but also between competition and collaboration, knowledge transfer and knowledge protection, and many others. Moreover, some of the operational paradoxes of the pre-digital age (see Westerman et al. 2014) that can be associated with open innovation, such as standardizing vs. empowering, controlling vs. innovating, and orchestrating vs. unleashing, can now be addressed in a digitalized world through ‘both-and’ approaches rather than trade-offs based on ‘either-or’ alternatives. We invite scholars to explore both how traditional open innovation paradoxes evolve and how new paradoxes emerge within a renewed open innovation research landscape. Moreover, although open innovation research has focused predominantly on paradoxes of learning and organizing, we know little about what Smith and Lewis (2011) have described as paradoxes of performing and belonging. Paradoxes of performing can emerge, for instance, in open innovation contexts at the intersection between the individual, the organizational, and the inter-organizational level, when individuals are called to perform different, often contradictory roles, while paradoxes of belonging reflect identity tensions between the individual and the collective, as individuals and groups seek both homogeneity and distinction. A multi-stakeholder view of open innovation also stimulates reflections on power dynamics in inter-organizational contexts, especially in purpose-driven initiatives where coordination and the model of authority are often unclearly defined. The role of marginalized actors, who are often beneficiaries of innovative solutions, and the contribution of firms that are called to partner a many-to-many stakeholder rather than a firm-centric game, raises questions in terms of power relations that are worth addressing.

Also, the systemic nature of the challenges that open innovation is called to address opens the doors to new paths that invite open innovation scholars to cross-fertilize open innovation studies with other fascinating perspectives. The role of open innovation in addressing environmental problems, for instance, can be explored by theoretical approaches that frame their analysis in system theories, such as social-ecological studies (Dentoni et al. 2021; Olsson et al. 2004). More specifically, the Schumpeterian view of entrepreneurship and innovation (advocated several times in the papers included in our sample), according to which entrepreneurs’ and innovators’ fervid distributed experimentation has, per se, positive consequences for the system’s performance, does not explain how innovation switches from the engine of (system-threatening) economic growth to being the engine of sustainable development that requires the preservation and (re)generation of social and environmental resources for the collective use (Ricciardi et al. 2021). In other words, a more comprehensive view of open innovation that considers the joint innovators as part of a social-ecological system characterized by many possibly hidden and nonlinear fragilities would inform about the possible negative disruptive consequences of creatively destructive actions.

Moreover, the recent association of open innovation with collective action (Mair and Gegenhuber 2021) may invite scholars to draw on the theory of the commons (Ostrom 1990) to explore the social dilemmas that emerge between individual and community interests. Indeed, when open innovation unfolds as collective action that involves multiple actors with different institutional logics, a social dilemma is presented to the community of innovators: they might choose short-term, personal benefits rather than constructing a common logic (Ansari et al. 2013), thus increasing the risk that the long-term collective benefit will be lost. Social dilemmas when open innovation mobilizes collective action can also be explained by drawing insights from game theory, another theory that is mentioned by the papers in our sample. Game theory can also prove to be useful to explore coopetition dynamics in open innovation. In this regard, Le Roy et al. (2018) contend that, together with network theory, resource-based theory, and capabilities-based theory, game theory contributes to an optimistic perspective or view of competition. In fact, one of the core postulates of game theory is that the best partner in a business is a competitor, which implies that both collaboration and competition with such a partner provide a suitable positive-sum game (Heiets et al. 2021).

To conclude, three reflections arise from our review in terms of theoretical developments in the field of open innovation studies. First, theories of the firm are still valuable, but not sufficient to explain open innovation in the face of the turmoil caused by ongoing transitions towards more digital and sustainable organizational and inter-organizational practices. Second, open innovation research would benefit from the combination of existing management theories, especially across levels. Finally, the third reflection is basically an open question: will existing theories be enough to explain open innovation in the future or should we focus our efforts on grounding new theorizing?

### Open innovation research methods and analytical techniques

Open innovation has been researched from a variety of methods and techniques. In the early stages of open innovation research, case studies were the predominant method because of their usefulness in illustrating successful cases and informing practitioners about best managerial practices (e.g., Chesbrough & Crowther 2006; Chesbrough 2007). Over the years, this method has been successfully reproposed to illustrate the role of open innovation in new domains such as sustainability (e.g., Bogers et al. 2020). Recently, research has also made use of case studies to shift the focus to the dark sides of open innovation, shedding light on the challenges and the possible drawbacks of open innovation (e.g., Bertello et al. 2021a; Stefan et al. 2022). Multiple case studies have been conducted likewise to carry on analyses between successful and unsuccessful cases (e.g., Grama-Vigouroux et al. 2020; Lazzarotti et al., 2013).

On the other hand, quantitative analyses have mainly focused on exploring the relationships between open innovation and firm performance (e.g., Ahn et al. 2015; Bianchi et al., 2016; Du et al. 2014; Greco et al. 2016), as well as the antecedents of open innovation at both the individual and organizational level (e.g., Bogers et al. 2018b; Naqshbandi & Tabche 2018). These studies either draw their hypotheses from close domains (e.g., inter-organizational collaboration, innovation projects) and apply them to open innovation empirical contexts, or develop research designs to test insights from open innovation case studies and conceptual papers. However, the most employed quantitative approaches in open innovation studies rely on conventional statistical (symmetrical) approaches such as regressions (e.g., Bogers et al. 2018b; Mention 2011; Papa et al. 2018) and structural equation modeling (e.g., Huang et al. 2013; Santoro et al. 2018) that may not adequately explain non-linear and complex real-world business phenomena. Linear statistical analyses with finite contextual factors could indeed suffer from prediction inaccuracy (Pappas and Woodside 2021). This prediction inaccuracy is significantly increased by digital and sustainability transformation processes that cause shifts in market circumstances and environmental conditions. Recently, organizational and management research has invoked the use of asymmetrical techniques that draw on the ontological assumptions of complexity theory, such as fuzzy-set qualitative comparative analysis (fsQCA) (Kumar et al. 2022). FsQCA is a statistically-informed configurational approach that aims to bridge and transcend the qualitative‒quantitative divide in social sciences (Ragin 2014). Research on open innovation still counts sparse contributions that have used complexity theory and fsQCA. This method could, however, be used increasingly in the future to depict the combinations of conditions that lead to the absence of an outcome, be it positive or negative. For instance, Peris-Ortiz et al. (2018) have used this method to explore the combination of factors that enable incremental innovation and foster radical innovation in open innovation contexts, focusing principally on human resources and organizational learning capabilities, while Kumar et al. (2022) have suggested applying fsQCA in open innovation studies to explore coopetition, trust and distrust dynamics, and human capital.

Changes in the methodologies adopted should also consider how open innovation practices are changing. For instance, the diffusion of digital platforms and online communities actually challenges traditional ways of collecting data and conducting field work. In this regard, the keyword netnography has emerged from the keyword co-occurrence analysis for the time period 2016‒2021. This emerging qualitative research method is already widely used in marketing studies (e.g., Pera et al. 2021). The term combines the words ‘internet’ or ‘network’ with ‘ethnography’ to highlight the role of online participant observation in exploring closely the dynamics of social interaction in contemporary digital communications contexts (Kozinets 2015). Its use could assume increasing relevance in exploring the role of users and customers in shaping open innovation practices such as new product development in an ever-increasing digitalized world (Bartl et al. 2016). Netnography, and, more generally, digital ethnography, could also represent a valuable tool for scholars who want to either extend offline data collection or explore social interaction dynamics in online contexts (Pink et al. 2015). In particular, this research method can be relevant to research online participatory contexts such as online hackathons and, more generally, crowdsourcing initiatives. These events, rapidly spread during the COVID-19 crisis, seem to be destined to become enduring models of innovation, even after the end of the pandemic, due to their capacity to gather at the same time different people from around the world (Bertello et al. 2021b). Ethnographies, be they digital or in person, would also contribute to exploring the temporal unfolding of open innovation activities, supporting a longitudinal perspective that is often lacking in open innovation studies. Indeed, open innovation studies have often prioritized variance theories, which refer to conceptual constructions that relate variables to one another, thus reducing time to a lag effect, or compressing it into variables, while process-based theorizing (i.e., ‘conceptual constructions that focus on the way in which phenomena emerge, evolve or terminate over time through activities and events’, see Cloutier & Langley 2020, p.3) is under-developed (Alam et al. 2022). Idea generation contexts such as hackathons, for instance, could be explored by paying attention to the subsequent phases of the innovation journey to understand how hackathon ideas can scale up over time and generate long-term impact (Mair and Gegenhuber 2021), while a linear conception of leveraging external sources as originally conceived in the early stages of open innovation research (West and Bogers 2014) might be further overcome by recursive process models that consider feedback loops and reciprocal interactions. Similarly, open innovation projects that require organizational change and digital transformation might be explored by paying attention to how different organizational structures, practices, and culture evolve and influence each other over time. Indeed, a process turn in open innovation studies might provide scholars with new conceptual tools to capture the complexity of an open innovation journey or an organizational re-design by considering chains of interconnected events, activities, and/or interactions in which the preceding events, activities, and/or interactions enable and/or constrain the following ones (see Langley et al. 2013).

Finally, in order to capture the complexity of open innovation dynamics in the face of sustainability challenges that are systemic by nature (Ferraro et al. 2015), we invite scholars to study open innovation practices by adopting the analytical technique of zooming in and zooming out, originally developed by Nicolini (2009) and, more recently, applied to sustainability and grand challenges studies by other scholars (see Jarzabkowski et al. 2019; Schad & Bansal 2018). By zooming into the organizing aspects of open innovation practices and then zooming out to the wider consequences of these practices, scholars would be able to capture the system-level consequences of open innovation in the face of grand challenges and to arrange organizational and inter-organizational practices accordingly. Another useful modeling tool to explore the system-level consequences of open innovation activities is system dynamics (see Richardson 2011). System dynamics is a valuable approach to understand the nonlinear behavior of complex systems and, therefore, to assess the social and environmental impact of open innovation practices over time by making use of stocks, flows, internal feedback loops, table functions, and time delays. Forliano et al. (2020), for instance, used this method to investigate the credit collection process of a municipally-owned company. By leveraging a system dynamic model, the authors moved beyond the traditional logic of linearity to capture the systemic, unintended, and delayed implications of decision-making activities related to the provision of public services and consider citizens as active stakeholders in the process of public value co-creation.

To stimulate a turn into practice-based and process-based approaches in open innovation studies, we draw on Weick’s intuition of translating nouns into verbs in organization studies (Weick 1969), and we introduce the concept of open innovating, inviting open innovation scholars to explore related phenomena by prioritizing processes over outcomes and activities over products.

## Conclusion

Open innovation has become a widely used concept in academia, industry, and policy-making in recent years (Bogers et al. 2018a). After its conceptualization (Chesbrough 2003), the open innovation model gradually spread among practitioners of large companies and high-tech sectors (Mortara and Minshall 2011; Parida et al. 2012) to then expand to a wide set of areas and domains, such as small and medium-sized enterprises (SMEs) (Santoro et al. 2020; Scuotto et al. 2017), low-tech firms (Bertello et al. 2021a; Dooley and O’Sullivan 2018), public organizations (Forliano et al. 2020; Randhawa et al. 2019; Schmidthuber et al. 2019) and online communities (Bertello et al. 2021b). Against this background, public authorities have aligned their policies with the open innovation imperatives to support the diffusion of open innovation practices across a wider audience. At the same time, the open innovation community of scholars has opened the doors to an ever-increasing number of scholars. Recently, open innovation practitioners have been called to confront ongoing societal transitions that are supposed to guide us towards a more sustainable and digital society. On the one hand, our future survival is endangered by natural disasters (Klein et al. 2019; Yang et al. 2018) and new (or renewed) complex, societal challenges that require collaborative and coordinated efforts from a variety of actors (Ferraro et al. 2015; George et al. 2016). On the other hand, the pervasiveness of digital artefacts is providing firms with the opportunity to connect multiple innovators, customize products, improve the efficiency of their processes, and achieve a broader market (Adamides and Karacapilidis 2020; Bertello et al. 2021c; Kraus et al. 2019; Nambisan et al. 2019; Talwar et al. 2021), but it also causes disruptions by challenging, for instance, the labor force’s existing skills as well as existing routines and practices (Balsmeier and Woerter 2019). This study aims to depict how the academic discourse on open innovation has evolved over the last 12 years (more precisely 2010‒2021) in the face of disruptive changes brought about by the transition to a more digital and sustainable society.

Despite being designed to contribute to clarify and advance research on open innovation in management, strategy, and organization studies, this work is not free of limitations.

First, as in any bibliometric analysis, the selection criteria imposed to improve the performance analysis (e.g. publication year, document type, language) limit the number of analyzable documents. For instance, the founder of the open innovation movement, Chesbrough, has written many books that are not anonymously peer-reviewed and, therefore, are excluded from the analysis. To mitigate this problem, in our discussion, we have also been inspired by seminal books to generate a more fruitful theoretical elaboration of the insights resulting from the bibliometric and content analysis.

Second, we conducted our analysis along two time periods. Although we rigorously explain in the methodology why the selected threshold is instrumental to the aim of this study, the criteria according to which we selected that threshold are still arbitrary. We invite scholars to explore longitudinally the evolution of open innovation studies by deploying other analyses that capture the historical evolution of this stream of research without necessarily identifying a priori specific time periods.

## Footnotes

1 In Table 2, the reference registers 2018 as the year of publication since the metadata used to run the analysis refers to the early access version.
